# Taxonomic and Phylogenetic Resolution of Novel Endophytic Arthrinium-like Fungi with an Updated Checklist of *Nigrospora* Species

**DOI:** 10.3390/life16061011

**Published:** 2026-06-16

**Authors:** Jutamart Monkai, Rungtiwa Phookamsak, Darbhe Jayarama Bhat, Danushka S. Tennakoon, Sinang Hongsanan, Toe Swe Zin Ei, Jianchu Xu, Saisamorn Lumyong

**Affiliations:** 1 Office of Research Administration, Chiang Mai University, Chiang Mai 50200, Thailand; mjutamart@gmail.com; 2 Department of Biology, Faculty of Science, Chiang Mai University, Chiang Mai 50200, Thailand; toeswezinei3081993@gmail.com; 3 Centre for Mountain Futures (CMF), Kunming Institute of Botany, Kunming 650201, China; phookamsak@mail.kib.ac.cn (R.P.); jxu@mail.kib.ac.cn (J.X.); 4 Honghe Center for Mountain Futures, Kunming Institute of Botany, Chinese Academy of Sciences, Honghe 654400, China; 5 Department of Economic Plants and Biotechnology, Yunnan Key Laboratory for Wild Plant Resources, Kunming Institute of Botany, Chinese Academy of Sciences, Kunming 650201, China; 6 Department of Botany and Microbiology, College of Science, King Saud University, Riyadh 11451, Saudi Arabia; bhatdj@gmail.com; 7 Biology Division, Vishnugupta Vishwavidyapeetam, Ashoke, Gokarna 581326, India; 8 Biology Centre of the Czech Academy of Sciences, Institute of Entomology, 37005 České Budějovice, Czech Republic; danushkasandaruwanatm@gmail.com; 9 Shenzhen Key Laboratory of Microbial Genetic Engineering, College of Life Sciences and Oceanography, Shenzhen University, Shenzhen 518060, China; sinang333@gmail.com; 10 The Center for International Forestry Research and World Agroforestry (CIFOR-ICRAF) China Program, World Agroforestry (ICRAF), Kunming 650201, China; 11 Center of Excellence in Microbial Diversity and Sustainable Utilization, Chiang Mai University, Chiang Mai 50200, Thailand; 12 Academy of Science, The Royal Society of Thailand, Bangkok 10300, Thailand

**Keywords:** *Amphisphaeriales*, *Apiospora*, *Apiosporaceae*, hyphomycetous asexual morph, new species, polyphasic approach, Sordariomycetes, taxonomy

## Abstract

Arthrinium-like fungi in the family *Apiosporaceae* are taxonomically complex and still require a thorough characterization despite recent phylogenetic reassessments. This study aimed to investigate the diversity and taxonomic position of endophytic Arthrinium-like fungi associated with *Itea japonica* and *I. riparia* in Thailand. Two fungal strains discovered from healthy stems of these hosts were characterized by integrative approaches including morphology, multi-locus phylogenetic analyses based on ITS, LSU, TEF1-α, TUB2 sequence data, and nucleotide base–pair comparisons. One isolate from *I. japonica* is introduced as *Nigrospora iteae* sp. nov. supported by distinct morphological traits, a well-resolved phylogenetic placement, and significant nucleotide difference from its closest relatives. The second isolate was identified as *Apiospora vietnamensis* and is reported herein as a new host record for *I. riparia* based on morphological congruence, a close phylogenetic relationship, and TUB2 nucleotide similarity with the type strain. In addition, a new species, *Apiospora fici*, originally described from dead leaves of *Ficus septica* in Taiwan, is reclassified based on updated phylogenetic analyses to clarify its taxonomic placement within *Apiosporaceae*. Furthermore, *Nigrospora wurfbainiae* nom. nov. is proposed as a replacement name for the later homonym *N. guangdongensis*. A summary of important morphological characteristics, host relationships, current distribution, and biological activities of *Nigrospora* species is provided. This study emphasizes the previously unrecognized fungal diversity within *Itea* hosts and offers new insights into species diversity and phylogenetic relationships within the *Apiosporaceae*.

## 1. Introduction

Arthrinium-like taxa are taxonomically characterized by the production of asexual Arthrinium-like basauxic conidiogenesis and sexual Apiospora-like ascospores in the family *Apiosporaceae* (*Amphisphaeriales*, *Xylariomycetidae*, *Sordariomycetes*), comprising genera *Apiospora*, *Arthrinium*, and *Nigrospora* [1,2,3,4]. Members of the *Apiosporaceae* occur across a wide range of hosts and habitats and function as pathogens of humans, animals, and plants, as well as saprophytes and endophytes [5,6,7]. The genus *Arthrinium* was introduced by Schmidt and Kunze [8] with the generic type, *A*. *caricicola*; *Apiospora* was later proposed by Saccardo [9], and is typified by *Ap. montagnei*; while *Nigrospora* was introduced by Zimmerman [10] with *N. panici* as the type species. Previously, *Apiospora* was considered as a synonym of *Arthrinium* based on its sexual morph [1]. However, Pintos and Alvarado [6] provided taxonomic resolution using multi-locus phylogenetic analyses, confirming *Apiospora*, *Arthrinium*, and *Nigrospora* as distinct genera.

The asexual morphologies of these three genera; *Apiospora*, *Arthrinium*, and *Nigrospora*, are extremely similar, particularly in their deeply pigmented conidia [11]. Conidia of *Apiospora* and *Nigrospora* are typically globose to subglobose in front view and lenticular in side view, while those of *Arthrinium* display variable shapes such as angular, curved, globose, fusiform, or polygonal ones [1,6,12]. Conidiogenesis of *Apiospora* and *Arthrinium* often involves two or more conidia or in clusters of conidia basauxically developed on each conidiogenous mother cell, whereas *Nigrospora* holoblastically produces a single conidium for each conidiogenous cell [1,4,5,11]. Consequently, it remains challenging to delimit Arthrinium-like taxa based solely on morphological characteristics. A combination of morphological observations and molecular phylogenetic studies is therefore essential for accurate taxonomic identification at both genus and species levels [5,12,13]. Multi-locus phylogenetic analyses based on the combination of ITS, LSU, TEF1-α, and TUB2 have substantially improved the species resolution of *Apiospora* and *Nigrospora* [4,5,6,7,12,13,14]. Nevertheless, the taxonomic position of several species remains uncertain and requires further studies due to incomplete sequence information and limited sampling [5,6,13,14].

Beyond their ecological importance, endophytic fungi represent intriguing and largely unexplored sources of natural products with diverse biological properties [15]. Numerous species of *Apiospora* and *Nigrospora* have been discovered as endophytes in a variety of plant hosts [1,5,16]. These isolates have yielded new secondary metabolites and exhibited promising biological activities, including the production of industrially relevant enzymes, anti-fungal agents, and antioxidants [17,18]. Studies on endophytes related to Arthrinium-like species has gained increasing attention due to their potential applications. However, the taxonomy and diversity of Arthrinium-like endophytic fungi in Thailand have been poorly investigated, particularly in association with traditional and medicinal plants. To address this gap, healthy stems of *Itea japonica* and *I. riparia* were collected from northern Thailand, from which two Arthrinium-like strains were isolated. The objective of this study is to clarify the taxonomic status of these new strains and related taxa through morphological characterization, multi-locus molecular analyses, and nucleotide sequence comparisons. Furthermore, a checklist of *Nigrospora* species is provided and discussed in terms of species diversity, host associations, geographical distribution, and biochemical properties.

## 2. Materials and Methods

### 2.1. Collection, Isolation and Morphological Observation

Asymptomatic tissues of *Itea japonica* and *I. riparia* were collected from Chiang Mai Province, Thailand. The stem part was disinfected and isolated for fungi using the protocol described by Monkai et al. [19]. For each host species, sixteen pieces of stems (5 mm^2^) were cut and sterilized with distilled water for 1 min, 70% ethanol for 30 s, 2% NaOCl for 30 s, air-dried, and placed on potato dextrose agar (PDA) plates. The plates were incubated at 25 °C in darkness for 1–2 days, and the single-hyphal tips were transferred onto fresh PDA plates. In total, 17 fungal strains were isolated from both hosts, of which two strains belonged to *Apiosporaceae*. Under growth conditions of 25 °C in darkness for 30 days, the fungal cultures sporulated and produced black conidial masses on PDA. Morphological observation was performed using a stereo microscope (Nikon SMZ800N, Tokyo, Japan) and a compound microscope equipped with a Nikon DS-Ri2 camera (Nikon Eclipse Ni U, Tokyo, Japan). Each structure, viz. conidiophores, conidiogenous cells, conidia, and sterile cells, was measured by the Tarosoft (R) Image Frame Work program version 0.9.7. Photo plates were prepared using Adobe Photoshop version 21.2.4 (Adobe Systems, San Jose, CA, USA). For long-term preservation, the cultures were deposited in the Culture Collection of Sustainable Development of Biological Resources Laboratory (SDBR-CMU), Faculty of Science, Chiang Mai University, Chiang Mai Province, Thailand and the Kunming Institute of Botany Culture Collection (KUNCC), Kunming, Yunnan Province, China. The herbarium materials (dried cultures) were prepared as mentioned in Monkai et al. [18] and deposited in the Herbarium of the Department of Biology (CMUB), Faculty of Science, Chiang Mai University, Chiang Mai Province, Thailand. The new species were registered for the Index Fungorum numbers [20].

### 2.2. DNA Extraction, PCR Amplification, and Sequencing

The fungal cultures were cultivated at 28 °C for ten days and the scraped mycelia were used to extract DNA using the Biospin Fungus Genomic DNA Extraction Kit (BioFlux, Hangzhou, China). The PCR amplification of specific ribosomal DNA regions was performed using four phylogenetic markers, including the internal transcribed spacers region of ribosomal DNA (ITS), the partial 28S large subunit nuclear ribosomal DNA (LSU), the translation elongation factor 1-α (TEF1-α) and beta-tubulin (TUB2) with the primer pairs, ITS5/ITS4, LR0R/LR5, EF1-728F/EF1-986R, and T1/Bt2b, respectively. The PCR reaction volume and thermal cycling programs for each phylogenetic marker were followed by Tian et al. [13] and Monkai et al. [14]. The annealing temperatures were set as 56 °C for ITS, LSU and TEF1-α, and 55 °C for TUB2. The PCR products were sequenced by TsingKe Company (Kunming, Yunnan Province, China).

### 2.3. Phylogenetic Analyses

The generated sequences were assessed for their quality using BioEdit version 7.0.5.3 [21] and assembled using SeqMan v. 7.0.0 (DNASTAR, Madison, WI, USA). The NCBI nucleotide BLAST web server was applied for the primary identification of similar taxa (www.ncbi.nlm.nih.gov/blast/; accessed on 1 October 2025). Reference sequence data were downloaded from the GenBank database according to relevant publications [22,23,24] (Table 1 and Table 2). The sequence dataset of each locus was aligned using the MAFFT v7.307 online version ([25]; https://mafft.cbrc.jp/alignment/server/, accessed on 1 October 2025) and trimmed manually using BioEdit version 7.0.5.3 [21]. The combined sequence data of ITS, LSU, TEF1-α, and TUB2 were used to generate phylogenetic trees of *Apiospora* species according to previous studies [6,13,14,24]. The combined sequence data of ITS, TUB2, and TEF1-α were subjected to phylogenetic analyses of *Nigrospora* species following previous studies [5,16,22,23]. The maximum likelihood (ML) and Bayesian inference (BI) analyses were carried out to infer the phylogenetic relationships of novel described taxa. Through the CIPRES Science Gateway platform V3.3 [26], RAxML-HPC2 on XSEDE (v.8.2.12) and MrBayes on XSEDE v.3.2.7a were selected as the analysis tools [27,28]. The nucleotide substitution model of GTRGAMMA with 1000 bootstrap iterations was arranged in ML analysis [28]. The evolutionary models for the BI analysis were assigned by MrModeltest v.2.3 [29]. For BI analysis of *Apiospora*, the best-fit model of ITS, LSU, TEF1-α and TUB2 datasets were set as GTR+I+G, GTR+I+G, GTR+I+G and HKY+I+G, respectively. For the BI analysis of *Nigrospora*, the best-fit model of the ITS, TUB2, and TEF1-α datasets was set as SYM+I+G, HKY+I+G, and GTR+I+G, respectively. To determine posterior probabilities (PPs), six synchronized Markov chains were implemented for 10,000,000 generations, with trees sampled every 1000th generation [30]. The burn-in phase represented by 25% trees were discarded and the retained 75% trees were used when the convergence diagnostic hit the stop value (below 0.01) [30]. The final trees were viewed in the FigTree v1.4.0 program [31] and graphically modified using Adobe Illustrator version 24.3 (Adobe Systems, San Jose, CA, USA). The new sequences were submitted for the GenBank accession numbers via the NCBI submission portal (https://submit.ncbi.nlm.nih.gov/, accessed on 1 November 2025).

## 3. Results

### 3.1. Phylogenetic Analyses

**Figure 1:**
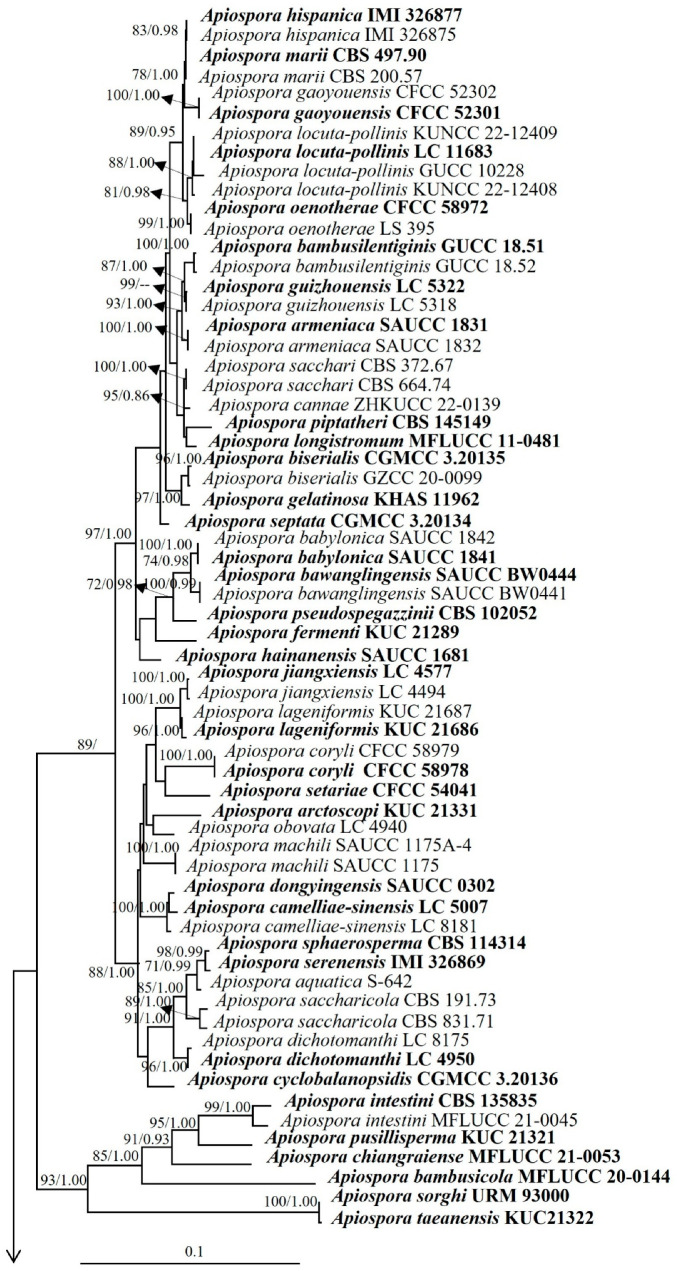

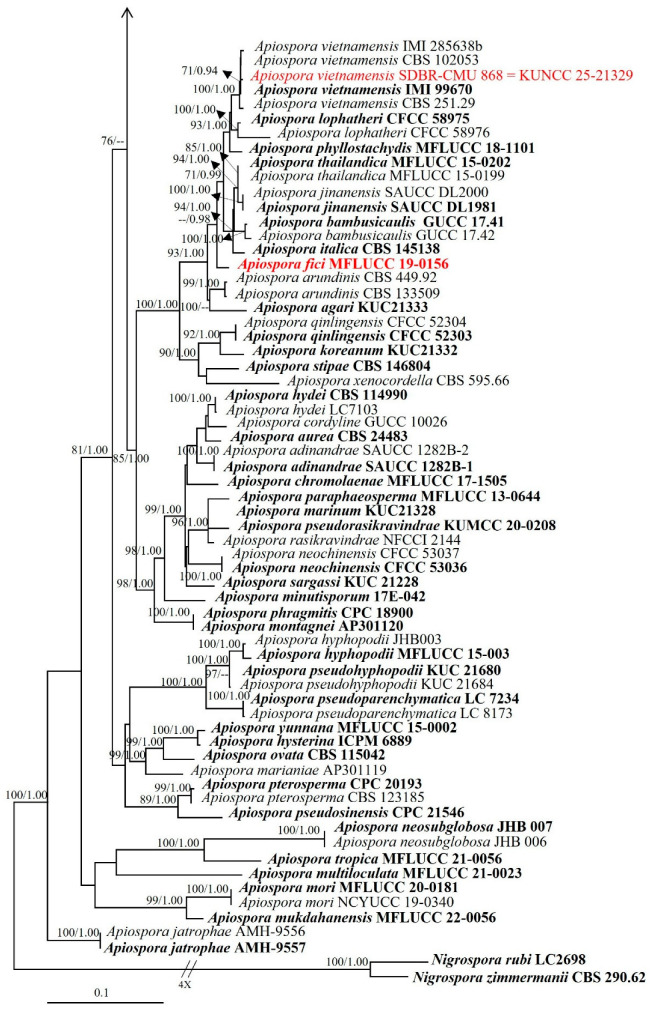
Phylogenetic tree inferred from RAxML analyses of combined ITS, LSU, TEF1-α, and TUB2 sequence alignments. ML bootstrap values ≥ 70% (left) and Bayesian inference posterior probability ≥ 0.90 (right) are given at the nodes (ML/BYPP). The tree is rooted to *Nigrospora rubi* (LC2698) and *N. zimmermanii* (CBS 290.62). Ex-type strains are in bold. New species and a new record are indicated in red.

The phylogenetic analysis of combined ITS, LSU, TEF1-α, and TUB2 sequence data comprised 124 taxa of *Apiospora* including the outgroup taxa, *Nigrospora zimmermanii* (CBS 290.62) and *N. rubi* (LC2698) (Figure 1). The integrated alignment dataset consisted of 3264 total characters (ITS: 1–632 bp, LSU: 633–1490 bp, TEF1-α: 1491–2329 bp, TUB2: 2330–3146 bp), including gaps. The RAxML analysis obtained a best-scoring tree with a final ML optimization likelihood value of −28330.285681. The matrix contained 1522 distinct alignment patterns, with 31.29% undetermined characters or gaps. Estimated base frequencies were recorded as follows: A = 0.236028, C = 0.255067, G = 0.252771, T = 0.256134; substitution rates AC = 1.158779, AG = 2.862804, AT = 1.039046, CG = 0.974162, CT = 4.197151, and GT = 1.000000. The gamma distribution shape parameter (*α* value) was equal to 0.244923. The average standard deviation of split frequencies for Bayesian analysis was 0.009990.

**Figure 2:**
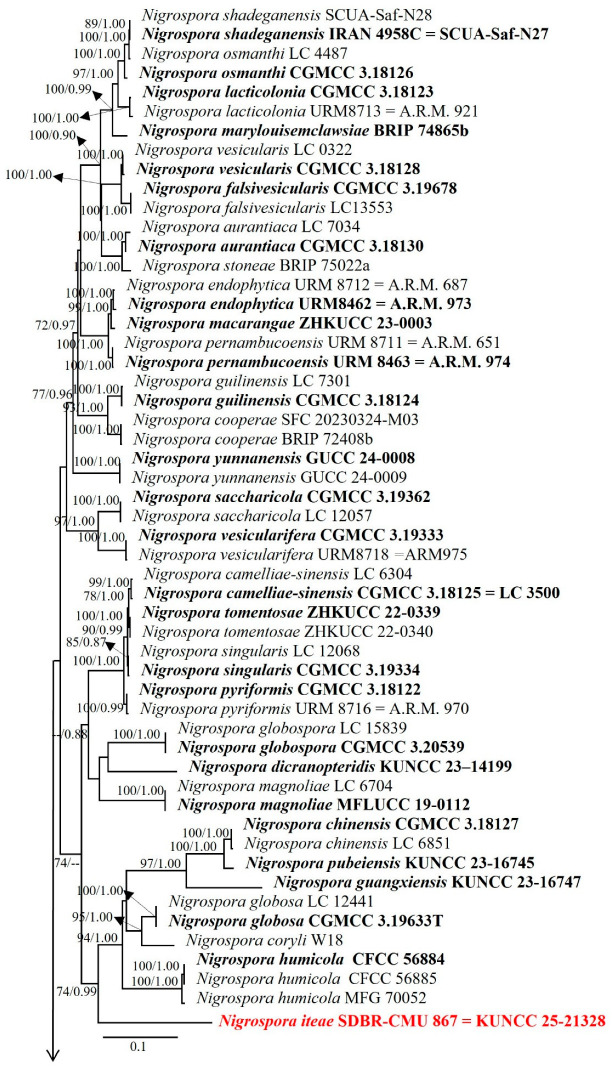

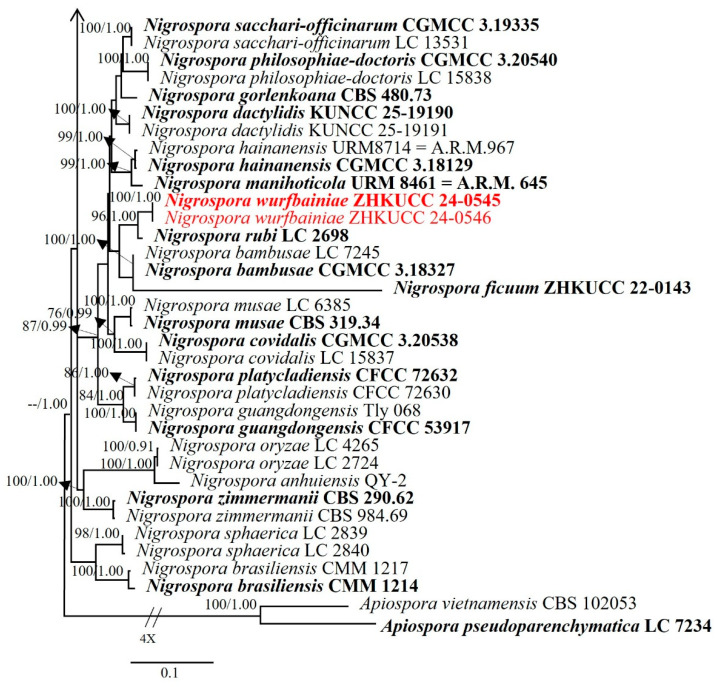
Phylogenetic tree inferred from RAxML analyses of combined ITS, TUB2, and TEF1-α sequence alignments. ML bootstrap values ≥ 70% (left) and Bayesian inference posterior probability ≥ 0.80 (right) are given at the nodes (ML/BYPP). The tree is rooted to *Apiospora vietnamensis* (CBS 102053) and *Ap. pseudoparenchymatica* (LC7234). Ex-type strains are in bold and a new species is indicated in red.

The phylogenetic analysis of combined ITS, TUB2, and TEF1-α sequence data comprised 80 taxa of *Nigrospora* including the outgroup taxa, *Apiospora vietnamensis* (CBS 102053) and *Ap. pseudoparenchymatica* (LC7234) (Figure 2). The integrated alignment dataset consisted of 1440 total characters (ITS: 1–545 bp, TUB2: 546–953 bp, TEF1-α: 954–1437 bp), including gaps. The RAxML analysis obtained a best-scoring tree with a final ML optimization likelihood value of −15780.707184. The matrix contained 831 distinct alignment patterns, with 9.83% undetermined characters or gaps. Estimated base frequencies were recorded as follows: A = 0.214482, C = 0.303732, G = 0.242489, T = 0.239296; substitution rates AC = 1.073038, AG = 2.998653, AT = 1.168011, CG = 0.923176, CT = 4.144400, and GT = 1.000000. The gamma distribution shape parameter (*α* value) was equal to 0.237948. The average standard deviation of split frequencies for Bayesian analysis was 0.009941.

Phylograms inferred from ML and BI analyses were topologically similar; therefore, the RAxML trees are presented as backbone trees. Phylogenetic analyses of combined ITS, LSU, TEF1-α, and TUB2 sequence data demonstrated that a new strain, SDBR-CMU 868, clustered with *Apiospora vietnamensis* IMI 99670 (ex-type), CBS 102053, CBS 251.29, and IMI 285638b, with high support (100% ML and 1.00 BYPP; Figure 1). Meanwhile, a strain, MFLUCC 19-0156, identified as *Ap. malaysiana* by Teennakoon et al. [32], constituted a distinct branch basal to *Ap. italica* (CBS 145138, ex-type) (0.98 BYPP: Figure 1). Therefore, the new species *Ap. fici* is proposed for *Ap. vietnamensis* MFLUCC 19-0156. The phylogenetic tree of *Apiospora* revealed that most species constituted a strongly supported clade (Figure 1). However, there are many species that were not well-resolved, such as *Ap. aquatica*, *Ap. arctoscopi*, *Ap. camelliae-sinensis*, *Ap. cannae*, *Ap. dongyingensis*, *Ap. fermenti*, *Ap. hispanica*, *Ap. marii*, *Ap. montagnei*, *Ap. phragmitis*, *Ap. Sacchari*, and *Ap. sargassi* (Figure 1). This requires additional phylogenetic studies with sufficient phylogenetic markers.

Phylogenetic analyses of combined ITS, TUB2, and TEF1-α sequence data revealed that *Nigrospora iteae* sp. nov. (SDBR-CMU 867) formed a well-resolved subclade sister to *N. humicola* (CFCC 56884, ex-type, CFCC 56885, MFG 70052) (74% ML, 0.99 BYPP: Figure 2). Meanwhile, *Nigrospora wurfbainiae* nom. nov. constituted an independent lineage sister to *N. rubi* (LC 2698, ex-type) with high support (100% ML and 1.00 BYPP; Figure 2). The phylogenetic tree of *Nigrospora* resulted in the separation of *Nigrospora* species in a well-resolved clade, except for *N. dicranopteridis*, *N. ficuum*, *N. gorlenkoana*, *N. osmanthi*, and *N. shadeganensis*, which require further taxonomic reassessment (Figure 2). Moreover, it should be noted that the TEF1-α sequences of *N. dactylidis* (PV626507, PV626508) and *N. globosa* (MK336056, MK336057) were not used in the analyses as their placements constituted a long branch and did not close with other species.

### 3.2. Taxonomy

#### 3.2.1. *Apiospora vietnamensis* (Hol.-Jech.) Pintos and P. Alvarado, Fungal Systematics and Evolution 7: 207 (2021)

**Figure 3:**
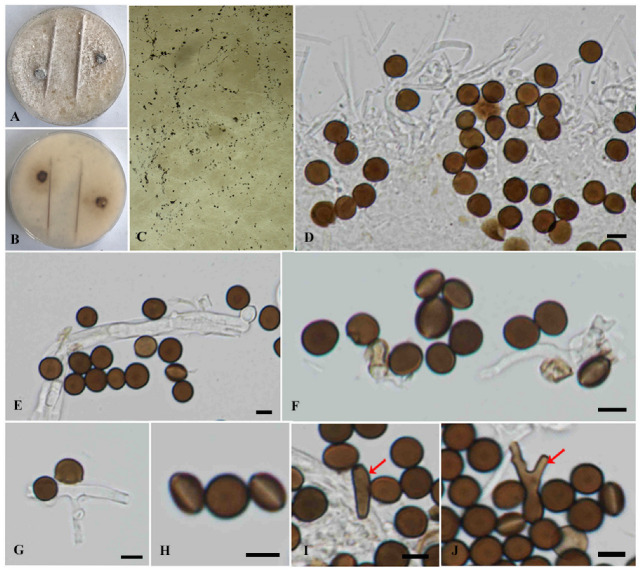
*Apiospora vietnamensis* (CMUB 40119). (**A**) Upper view of colonies on PDA. (**B**) Reverse view of colonies on PDA. (**C**) Hyphae and conidial masses. (**D**–**G**) Conidiogenous cells and conidia. (**H**–**J**) Conidia. (**I**,**J**) Sterile cells (indicated at arrows). Scale bars: (**D**–**J**) = 5 μm.

Index Fungorum number: IF837737; Figure 3.

*Basionym: Nigrospora vietnamensis* Hol.-Jech., Česká Mykologie 17(1): 19 (1963)

*Synonyms: Arthrinium euphorbiae* M.B. Ellis, Mycological Papers 103: 6 (1965)

*Arthrinium malaysianum* Crous, IMA Fungus 4(1): 144 (2013)

*Arthrinium vietnamense* (Hol.-Jech.) Mei Wang and L. Cai [as ‘vietnamensis’], Persoonia 39: 139 (2017)

*Apiospora euphorbiae* (M.B. Ellis) X.G. Tian and Tibpromma, Life 11(no. 1071): 17 (2021)

*Apiospora malaysiana* (Crous) Pintos and P. Alvarado, Fungal Systematics and Evolution 7: 206 (2021)

*Apiospora magnispora* H.J. Zhao, Manawas. and W. Dong, Current Research in Environmental and Applied Mycology 13(1): 9 (2023)

*Endophytic* on healthy stems of *Itea riparia*. Sexual morph: Undetermined. Asexual morph: Sporulated on PDA in 30 days of incubation at 28 °C, visible as black, effuse conidial mass, on white, aerial mycelium. *Hyphae* 1–2 μm diam., smooth, hyaline, branched, septate. *Conidiophores* reduced to conidiogenous cells. *Conidiogenous cells* 3.5–7 × 2.5–4 μm ($\bar{x}$ = 4.5 × 3 μm, *n* = 10), basauxic, aggregated in clusters on hyphae, hyaline to pale brown, doliiform to ampulliform or clavate. *Conidia* 5–7 × 4–6 μm ($\bar{x}$ = 6 × 5 μm, *n* = 30), pale brown to brown, aseptate, globose to subglobose in surface view, lenticular in side view, with a pale equatorial slit, smooth-walled. *Sterile cells* 8–15 × 2.5–3.5 μm ($\bar{x}$ = 10.5 × 3 μm, *n* = 5), brown, elongated ellipsoidal to clavate, branched.

Culture characteristics: Colonies on PDA reached at 9 cm diam. in 10 days at 28 °C, flat, fluffy, spreading with abundant aerial mycelia, edge entire, white to pale yellowish, becoming black with age, in reverse, pale yellowish and light gray in the center; not producing pigmentation.

Material examined: THAILAND, Chiang Mai Province, Mae Rim District, on living stems of *Itea riparia (Iteaceae)*, 10 July 2023, J. Monkai, IT83 (CMUB 40119, holotype), ex-type living culture, SDBR-CMU868 = KUNCC 25-21329, dried culture permanently preserved in a metabolically inactive state.

Known hosts: *Bambusa textilis* [33], Bats [34], *Cinnamomum camphora* [1], *Citrus sinensis* [35], *Euphorbia* [36], *Itea riparia* [this study], *Macaranga hullettii* [1], and *Termitomyces clypeatus* [37].

Distribution: Czech Republic [35], China [33,34], India [37], Malaysia [1], Thailand [this study], and Zambia [36]. Additional distributions that are listed in Global Biodiversity Information Facility (GBIF) include Brazil, Indonesia, Portugal, the United Republic of Tanzania, the United Kingdom, and Vietnam [38].

Notes: The nucleotide BLAST search of ITS region indicated that *Apiospora vietnamensis* (SDBR-CMU868) is similar to *Ap. malaysiana* isolate MFE2 (99.83%), Fungal sp. isolate N30 (99.83%) and *Fungal* sp. E15022H (99.83%). The nucleotide BLAST search of LSU region indicated that *Ap. vietnamensis* (SDBR-CMU868) is similar to *Ap. malaysiana* CBS 102053 (98.44%), *Apiospora* sp. strain KUMCC 21-0429 (98.44%), and *Ap. chromolaenae* culture MFLUCC:17-1505 (98.44%). The nucleotide BLAST search of TUB2 region indicated that *Ap. vietnamensis* (SDBR-CMU868) is similar to the *Arthrinium euphorbiae* strain: IMI 285638b (98.87%), *Ar. malaysianum* CBS 102053 (99.75%) and *Ar. euphorbiae* strain ZHKUCC 22-0001 (99.72%). The nucleotide BLAST search of TEF1-α region indicated that *Ap. vietnamensis* (SDBR-CMU868) is similar to *Ar. malaysianum* CBS 102053 (99.33%), *Ap. arundinis* isolate CC.XQDXG283.1 (98.06%), and *Apiospora* sp. QZ-2024a isolate CC.BYG75.1 (97.80%).

*Apiospora vietnamensis* was initially isolated as a saprobe on *Citrus sinensis* fruit and identified as *Nigrospora vietnamensis* [35]. Wang et al. [5] then moved it to *Arthrinium* before reclassifying it under *Apiospora* [6]. Wang et al. [38] synonymized *Ap*. *euphorbiae*, *Ap*. *malaysiana*, and *Ap*. *magnispora* under *Ap. vietnamensis* based on morphological and phylogenetic congruence. In our phylogenetic analysis, the new strain (SDBR-CMU868) clustered in a single subclade with the type and other strains of *Ap. vietnamensis*, with high support (100% ML and 1.00 BYPP; Figure 1). The base–pair comparison between our new strain (SDBR-CMU868) and the type strain, *Ap. vietnamensis* (IMI 99670) revealed 0.2% (1/570), 0.1% (1/802), and 0.3% (1/312) bp differences in ITS, LSU, and TUB2, respectively.

Morphological characteristics of our new strain (SDBR-CMU868) were compared with *Ap. vietnamensis* (both ex-type and other strains) (Table 3). Our new strain (SDBR-CMU868) is similar to the type strain of *Ap. vietnamensis* (IMI 99670) in having pale brown to dark brown, globose conidia of overlapping size (5–7 × 4–6 vs. 5–6 × 3–4 μm) [35] (Table 3). However, the conidiophores of the new strain are reduced to conidiogenous cells that differ from those in the type strain, which have hyaline septa and short, branched structures [35] (Table 3). The new strain has conidiogenous cells that are grouped in clusters on hyphae, hyaline to pale brown, doliiform to ampulliform, or clavate, resembling the strain CBS 102053, which was previously identified as *Ap. malaysiana* [1] (Table 3). Other strains of *Ap. vietnamensis* did not exhibit sterile cells, with the exception of IMI 285638b, which is tentatively identified as *Ap. euphorbiae* [1] (Table 3). Even though there were some morphological differences between *Ap. vietnamensis* strains, environmental conditions may have contributed to this effect, leading to unreliable evidence for species delimitation [14,38]. Therefore, based on morphology, phylogeny, and nucleotide pairwise differences, this novel strain is identified as *Ap. vietnamensis*. This is the first report of this species on the host plant, *Itea* and in Thailand.

#### 3.2.2. *Apiospora fici* Monkai, Tennakoon, Phookamsak, Bhat & S. Lumyong, sp. nov.

**Figure 4:**
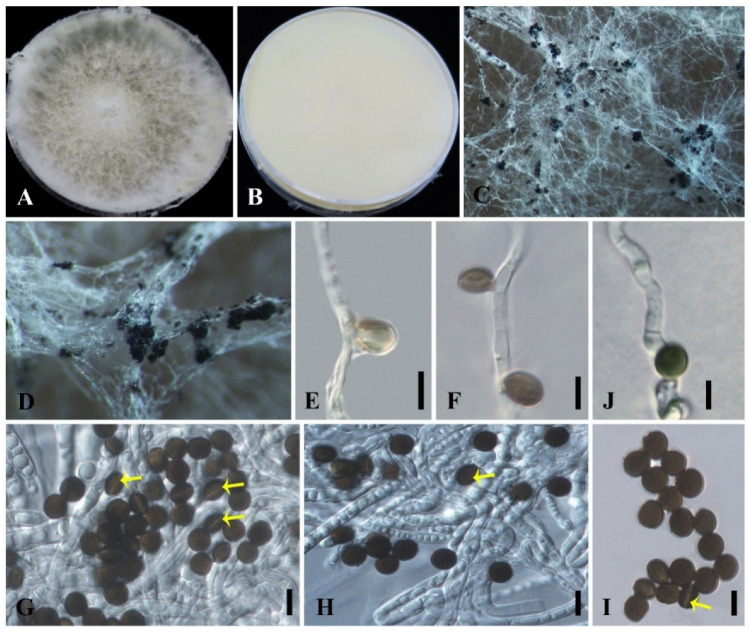
*Apiospora fici* (NCYU 19-0342, holotype): (**A**) Upper view of colonies on PDA. (**B**) Reverse view of colonies on PDA. (**C**,**D**) Conidial mass forming on PDA. (**E**,**F**) Conidiophores with conidiogenous cells bearing conidia. (**G**–**I**) Conidia (arrows shows germ-slit). (**J**) A germinating conidium. Scale bars: (**E**–**I**) = 5 µm.

Index Fungorum number: IF905202; Figure 4.

Etymology: Referring to the host genus, *Ficus* from which the holotype was collected.

Holotype: NCYU 19-0342

*Saprobic* on dead leaves of *Ficus septica*. Sexual morph: Undetermined. Asexual morph: Sporulated on PDA after a 3-week incubation period in the dark, at 25 °C, visible as black, effuse conidial mass, on white, aerial mycelium. *Hyphae* 1.5–2.5 μm diam., smooth to asperulate, hyaline to brown, branched, septate, thin-walled. *Sporodochia* dark, dense, dry, velvety *Conidiophores* up to 30 μm long, 3–6 μm wide, aggregated in sporodochia, smooth, hyaline to light brown, usually unbranched. *Conidiogenous cells* 5–7 × 3–5 µm ($\bar{x}$ = 5.8 × 3.9 μm, *n* = 20), basauxic, smooth to finely roughened, hyaline to pale yellow subcylindrical to doliiform. *Conidia* 5–6.5 × 4–5.5 µm ($\bar{x}$ = 5.5 × 4.5 μm, *n* = 30), dark brown, globose to subglobose, smooth-walled, with a truncate basal scar, with a longitudinal, hyaline, thin, germ-slit [32].

Culture characteristics: Colonies on PDA, 25–30 mm diam. after 3 weeks, colonies from above: medium dense, cottony, entire edge, white to cream at margin, light brown to gray at center; reverse: white to cream at margin, cream at center.

Material examined: CHINA, Taiwan, Chiayi, Ali Shan Mountain, Fanlu Township area, Dahu forest, on dead leaves of *Ficus septica* (*Moraceae*), 20 June 2019, D. S. Tennakoon, XP046 (NCYU 19-0342, holotype), ex-type living culture, MFLUCC 19-0156, NCYUCC 19-0190.

Notes: During our phylogenetic investigation, we found that the strain MFLUCC 19-0156 was not clustered in the same clade as the type strain of *Ap. vietnamensis* (IMI 99670), but formed an independent lineage within *Apiospora* (0.98 BYPP; Figure 1). Tennakoon et al. [32] isolated this strain from dead leaves of *Ficus septica* and identified it as *Ap. malaysiana* (current name: *Ap. vietnamensis*) based on morphology and phylogeny. This strain produces dark brown, smooth-walled, globose to subglobose conidia with an overlapping size range with the type strain *Ap. vietnamensis* (5–6.5 × 4–5.5 vs. 5–6 × 3–4 μm) [5,35]. The base–pair comparison between the strain MFLUCC 19-0156 and the type strain *Ap. vietnamensis* (IMI 99670) revealed 0% (0/570) and 0.1% (1/811) bp differences in ITS and LSU. The nucleotide comparison of TUB2 was not possible due to the lack of sequence data for the strain MFLUCC 19-0156. Since TEF1-α sequence data for *Ap. vietnamensis* (IMI 99670, ex-type) are absent, pairwise nucleotide was compared with *Ap. vietnamensis* (CBS 102053), resulting in 6.3% (27/427) bp differences. Thus, based on the distinct phylogeny and significant nucleotide difference of TEF1-α sequence data, we redescribe this strain and propose it as a new species, *Apiospora fici*.

#### 3.2.3. *Nigrospora iteae* Monkai, Phookamsak, Bhat & S. Lumyong, sp. nov.

**Figure 5:**
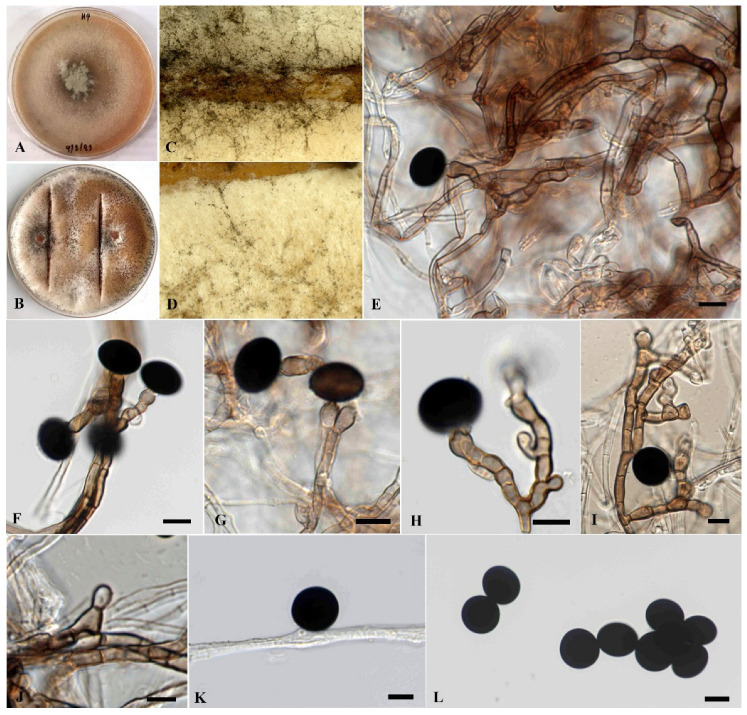
*Nigrospora iteae* (CMUB 40118, holotype). (**A**) Upper view of colonies on PDA. (**B**) Reverse view of colonies on PDA. (**C**,**D**) Hyphae and conidial masses. (**E**–**H**) Conidiophores, conidiogenous cells and conidia. (**I**,**J**) Developing conidiogenous cells (**K**–**L**) Conidia. Scale bars: (**E**–**L**) = 10 μm.

Index Fungorum number: IF904628; Figure 5.

Etymology: Referring to the host genus, *Itea* from which the holotype was collected.

Holotype: CMUB 40118

*Endophytic* on healthy stems of *Itea japonica*. Sexual morph: Undetermined. Asexual morph: Sporulated on PDA during 30 days of incubation at 28 °C, with abundant black effuse conidial mass. *Hyphae* 2–3 μm diam., smooth, hyaline to pale brown, branched, septate. *Conidiophores* 3.5–5.5 μm. diam., macro- to semi-micronematous, aggregated in clusters on hyphae, brown, branched, septate, straight or flexuous. *Conidiogenous cells* 5–13 × 4.5–8 μm ($\bar{x}$ = 9 × 6 μm, *n* = 30), holoblastic, discrete, determinate, brown, ampulliform to clavate. *Conidia* 15.5–21.5 × 12.5–19 μm ($\bar{x}$ = 19 × 16 μm, *n* = 30), solitary, black, velvety, aseptate, globose to subglobose or dorsi-ventrally compressed, smooth-walled.

Culture characteristics: Colonies on PDA reached a 9-cm diam. in 10 days at 28 °C, and were flat, floccose at the center with gray aerial mycelia, edge entire, initial white, becoming black with age, in reverse, orange brown; with pale orange pigment production.

Material examined: THAILAND, Chiang Mai Province, Hang Dong District, on living stems of *Itea japonica (Iteaceae)*, 7 February 2023, J. Monkai, IT31 (CMUB 40118, holotype), ex-type living culture, SDBR-CMU867 = KUNCC 25-21328, dried culture permanently preserved in a metabolically inactive state.

Notes: The nucleotide BLAST search of ITS region indicated that *Nigrospora iteae* (SDBR-CMU867) is very similar to *Nigrospora* sp. isolate KoRLI046022 (99.82%), *Nigrospora* sp. strain CMRP4535 (99.64%), and *Nigrospora* sp. strain CMRP4591 (99.64%). The nucleotide BLAST search of TUB2 region indicated that *N. iteae* (SDBR-CMU867) is similar to *N. pyriformis* strain LC3099 (95.82%), *Nigrospora* sp. 2 MW-2017 strain LC6704 (95.42%), and *N. pyriformis* strain LC2694 (95.40%). The nucleotide BLAST search of the TEF1-α region indicated that *N. iteae* (SDBR-CMU867) is similar to *Nigrospora* sp. strain ZHKUCC 24-0546 (89.07%), *Nigrospora* sp. strain ZHKUCC 24-0545 (89.07%), and *N. cooperae* strain BRIP 72440a (88.45%).

In our phylogeny, *Nigrospora iteae* formed a distinct lineage which is closely related to *N. humicola* (74% ML, 0.99 BYPP; Figure 2). Morphologically, *N. iteae* resembles *N. humicola* in having brown, branched, septate conidiophores and brown ampulliform conidiogenous cells [22]. However, *N. iteae* differs from *N. humicola* by its larger conidia (15.5–21.5 × 12.5–19 vs. 12.5–23.5 × 9.5–16) [22]. The base–pair comparison between *N. iteae* (SDBR-CMU867) and *N. humicola* (CFCC 56884) revealed 3.7% (19/518), 11.6% (43/372), and 23.8% (108/457) bp differences in ITS, TUB2, and TEF1-α. Thus, we identified this strain as a new species of *Nigrospora*.

#### 3.2.4. *Nigrospora wurfbainiae* Monkai, Hongsanan, Bhat and S. Lumyong, nom. nov.

Index Fungorum number: IF905203.

Replaced synnonym: *Nigrospora guangdongensis* Y.X. Li & Doilom [as ‘guangdongense’], Mycosphere 16(2): 117 (2025)

Etymology: Referring to the host genus, *Wurfbainia* from which the holotype was collected.

Holotype: MHZU 24–0242

Descriptions–see Hongsanan et al. [39]

Material examined: CHINA, Guangdong Province, Yangjiang City, 23°11′39” N; 113°21′22” E, on asymptomatic leaves of *Wurfbainia villosa* (*Zingiberaceae*), 1 October 2021, C.F. Liao, (MHZU 24–0242, holotype), ex-type living culture ZHKUCC 24–0545; *ibid*., living culture ZHKUCC 24–0546.

Notes: *Nigrospora guangdongensis* was introduced by Tian et al. [40], isolated from the needles of *Cunninghamia lanceolata* in Guangdong Province, China. Later, Hongsanan et al. [39] introduced a new species, named *N. guangdongense*, from asymptomatic leaves of *Wurfbainia villosa* (*Zingiberaceae*), which was also collected from Guangdong Province, China. The latter name is illegitimate due to the homonymy under Art. 53.1 of the ICN [41]. Thus, we proposed the name *N. wurfbainiae* as the replacement.

This species is characterized by hyaline, globose to subglobose to ampulliform conidiogenous cells, aseptate, smooth-walled, solitary, dark brown to black, subglobose to globose conidia [39]. Phylogenetic analyses of a combined ITS, TUB2, and TEF1-α indicate that *N. wurfbainiae* formed a monophyletic clade sister to *N. rubi* (100% ML, 1.00 BYPP; Figure 2). The base–pair comparison between *N. wurfbainiae* (ZHKUCC 24–0545) and *N. rubi* (LC2698) revealed 0% (0/486), 8.7% (32/367), and 0.6% (3/489) bp differences in ITS, TUB2, and TEF1-α. The evidences of morphology, phylogeny, and significant nucleotide difference in TUB2 sequence supported it as a separate species.

## 4. Discussion

This study provides taxonomic novelties and updates of Arthrinium-like species based on our new collections from *Itea* in Thailand together with previously reported taxa. With the application of polyphasic approaches, the number of *Apiospora* species has increased steadily in recent years [4,14,42,43,44]. Despite these advances, species identification remains challenging, and certain species complexes cannot yet be fully resolved [14,43]. In this study, phylogenetic analyses based on a combined ITS-LSU-TEF1-α-TUB2 sequence dataset demonstrated a close relationship between the new strain, SDBR-CMU 868, isolated as an endophyte from *Itea riparia*, and *Ap. vietnamensis* (IMI 99670, ex-type) (Figure 1). Morphological characteristics and nucleotide sequence data (ITS, LSU, and TUB2) showed only insignificant differences between the two strains. Based on this evidence, we identified the new strain as *Ap. vietnamensis*. Notably, phenotypic variation was observed among strains of *Ap. vietnamensis* (Table 3). Consistent findings have also been reported in other *Apiospora* species, such as *Ap. locuta-pollinis*, *Ap*. *pseudoparenchymatica*, and *Ap*. *yunnana*, highlighting their intraspecific variation, which is likely influenced by environmental factors such as temperature, elevation, growth media, substrates, and hosts [14,38].

Moreover, our phylogenetic investigation provides new insight into the taxonomy of *Apiospora*. The previously described strain MFLUCC 19-0156, a saprobe isolated from *Ficus* in Taiwan [32], is herein re-identified as a novel species since its phylogenetic placement is clearly separated from *Ap. vietnamensis* (=*Ap. malaysiana*). Furthermore, the phylogenetic placements of several species were not clearly resolved (Figure 1). The absence of TEF1-α and TUB2 sequence data in some strains, especially the type strains (i.e., *Ap. aquatica*, *Ap. hispanica*, *Ap. locuta-pollinis*, *Ap. marii*, *Ap. rasikravindrae*, *Ap. vietnamensis* and *Ap. yunnana*), may have affected their phylogenetic resolution as these loci are considered essential for species delineation [6,13,14,43]. Therefore, the taxonomic status of unresolved species requires a re-examination of their type specimens and further phylogenetic studies using additional markers such as TEF1-α, TUB2, and RPB2.

The geographical distribution and host-substratum diversity of *Apiospora* species were comprehensively compiled and visualized by Monkai et al. [14] and Wang et al. [38] Furthermore, the ecological modes and occurrence of sexual and asexual morphs in *Apiospora* were summarized based on available morphological and molecular evidence [43]. According to these reports and other recent studies, *Apiospora* species are distributed worldwide in subtropical, tropical, and temperate regions and exhibit different lifestyles as human and plant pathogens, endophytes, and saprobes [6,14,24,43,44]. Members of *Poaceae*, especially the bamboo species, represent their primary hosts [14,24,45,46]. To date, more than 200 *Apiospora* species have been reported, approximately half of which were described from China based on the USDA database (https://fungi.ars.usda.gov/, assessed on 1 June 2026) [14,20,24,45]. However, Wang et al. [38] indicated that the highest number of occurrence records for *Apiospora* was found in the United States, the United Kingdom, and China, respectively, according to the GBIF database (https://doi.org/10.15468/dl.dx2b4u, assessed on 19 January 2026). Although asexual and sexual morphs were documented in *Apiospora*, most species predominantly produce asexual morphs [43,46]. Notably, the sexual morph has been observed mainly in species exhibiting a saprobic lifestyle [43].

Furthermore, our study introduces a new species, *Nigrospora iteae*, isolated as an endophyte from *Itea japonica* in Thailand based on its monophyletic placement (Figure 2) and distinct morphology characterized by brown conidiophores and pale orange pigment production on PDA. We also provide nomenclatural modifications within *Nigrospora*. A replacement name, *Nigrospora wurfbainiae*, was erected to avoid the homonymy for *N. guangdongensis*. Our updated phylogeny, coupled with significant nucleotide variation in the TUB2 sequence, confirm it as a distinct species. The combined sequence data of ITS-TEF1-α-TUB2 provide an effective approach to delineate the *Nigrospora* species, as shown both in our study and previous studies [7,22,23,47,48]. In particular, a significant genetic divergence in one locus or both TEF1-α and TUB2 provides strong evidence for species delimitation in *Nigrospora* [5,22,23,47,48]. Nevertheless, we excluded the TEF1-α sequences of *N. dactylidis* and *N. globosa* from our phylogenetic analyses as they were phylogenetically inconsistent with other *Nigrospora* taxa. These sequence data need to be re-examined to determine whether they were misidentified. Moreover, due to uncertain placement and the lack of molecular data from type specimens for several species, future phylogenetic reevaluation using additional markers such as RPB2 and ACT are necessary.

In this regard, a checklist of *Nigrospora* species and their relevant data was summarized based on the USDA Fungus-Host database (https://fungi.ars.usda.gov/, assessed on 1 June 2026), Index Fungorum (https://indexfungorum.org/Names/Names.asp, accessed on 1 June 2026), recent publications, and this study (Table 4). At present, 62 *Nigrospora* species are accepted, of which 53 species have molecular data available (Table 4). However, *Nigrospora anhuiensis* and *N. coryli* are invalid since their holotype was not designated [49,50], resulting in a nom. inval., under Art. 40.1 of the International Code of Nomenclature for Algae, Fungi, and Plants (ICN) [41]. Morphologically, *Nigrospora* species have been mainly described from asexual structures produced on artificial media, most commonly potato dextrose agar (PDA), but also on synthetic nutrient-poor agar (SNA) and oatmeal agar (OA) (Table 4). Additionally, three recently proposed species, e.g., *N. marylouisemclawsiae*, *N. mercuriadeae*, and *N. stoneae* [51,52,53], lack sufficient morphological documentation, underscoring the importance of an integrative taxonomy that incorporates both morphology and phylogeny.

In terms of diversity and ecological distribution, *Nigrospora* species have been extensively isolated from living plants as endophytes and pathogens [5,16,23,66] (Table 4). A few species have been isolated from soil and air, such as *N. sphaerica*, which is an opportunistic causal agent of human diseases [95,96]. Host associations are varied, with some species appearing to be specific to a single plant (e.g., *N. bambusae*, *N. brasiliensis*, *N. falsivesicularis*, *N. saccharicola*, and *N. vesicularis*) and some species with a diverse range of plants (e.g., *N. aurantiaca*, *N. camelliae-sinensis*, *N. chinensis*, *N. lacticolonia*, *N. oryzae*, *N. osmanthi*, *N. pyriformis*, and *N. sphaerica*). The most common occurrences of plant hosts are *Camellia* spp., *Musa* spp., and members of *Poaceae* such as *Bambusoideae*, *Oryza sativa*, *Saccharum officinarum*, and *Zea mays* [5,20,46,66] (Table 4). The geographical distribution of the *Nigrospora* species is widespread, occurring mainly in Asia, especially China, and, to a lesser extent, in South America and Australia [20,45] (Table 4). The three species, *N. oryzae*, *N. sacchari*, and *N. sphaerica*, have a widespread distribution [5]. Wang et al. [5] indicated that *Nigrospora* species tend to evolve from having a wide to a narrow host range, which is related to the evolution of fungal pathogens. The recent discovery of several new *Nigrospora* species, with endophytic and pathogenic modes in specific plant hosts, highlights their lifestyle adaptations ([22,39,45,46,47,65,84,92], this study). Hence, it is important to evaluate the species diversity of *Nigrospora* from both symptomatic and asymptomatic tissues of important economic crops and ornamental plants for effective disease control and management strategies.

Regarding chemical potential, various species (viz. *Nigrospora aurantiaca*, *N. chinensis*, *N. guilinensis*, *N. lacticolonia*, *N. oryzae*, *N. sacchari*, and *N. zimmermanii*) were screened for productions of secondary metabolites resulting in enormous industrial and pharmaceutical value such as anti-cancer, anti-inflammatory, antimicrobial, antioxidant, insecticidal, and plant-growth enhancement [17,73,86,87,93] (Table 4). Many bioactive compounds were reported from endophytic terrestrial plants and marine environments [17]. However, numerous strains of *Nigrospora* reported in the literature were not identified at the species level and thus were not included in our summary. Further research is needed to explore the untapped chemical diversity and potential applications of additional *Nigrospora* species.

The genus *Itea* comprises medicinal and ornamental plants known as interesting sources of natural products such as rare sugar [97,98]. Thus, fungal endophytes isolated from *Itea* may serve as effective biofactories for the synthesis of bioactive metabolites. Recent taxonomic investigations of endophytic fungi associated with *Itea* species have revealed 11 novel pestalotioid taxa, including two new species and three new host records [19]. Expanding on this research, this study aims to explore the undescribed fungal diversity associated with this host genus. Our results support the introduction of *Nigrospora iteae* as a new species from *Itea japonica* and report *Apiospora vietnamensis* from *I. riparia* as a new host record for this genus. So far, only one *Nigrospora* species *N. chinensis* has been reported, from *Itea* sp. in Jiangxi Province, China, although the type of association, whether pathogenic or endophytic, was not specified [5]. Thus, this is the second report of *Nigrospora* species from *Itea*.

## 5. Conclusions

This study provides comprehensive morphological observations, updated phylogenetic analyses, and information on host relationships, geographical distribution, and biological potential, which together enhance the knowledge of Arthrinium-like fungi. This study resolves nomenclatural issues, clarifies species boundaries, and broadens the host distribution of *Apiospora* and *Nigrospora* through combined morphological comparisons and multi-locus phylogeny. The key morphological characteristics and summarized information concerning *Nigrospora* species provided in this study (Table 4) will therefore be a valuable reference for future taxonomic studies and applied research. Nevertheless, several limitations remain, including unresolved phylogenetic placements, a limited number of collections, and the lack of functional and ecological investigations. Future studies incorporating broader sampling, additional molecular markers, and functional experiments are therefore necessary to further improve our understanding of the diversity, ecology, and biological significance of these fungi.

## Figures and Tables

**Table 1:** Information of fungal taxa used for phylogenetic analyses of *Apiospora* in this study.

Species Name	Strain Codes	GenBank Accession No.			
		ITS	LSU	TEF1-α	TUB2
*Apiospora adinandrae*	SAUCC 1282B-1 ^T^	OR739431	OR739572	OR753448	OR757128
*Ap. adinandrae*	SAUCC 1282B-2	OR739432	OR739573	OR753449	OR757129
*Ap. agari*	KUC 21333 ^T^	MH498520	N/A	MH544663	MH498478
*Ap. aquatica*	S-642	MK828608	MK835806	N/A	N/A
*Ap. arctoscopi*	KUC 21331 ^T^	MH498529	N/A	MN868918	MH498487
*Ap. armeniaca*	SAUCC DL1831 ^T^	OQ592540	OQ615269	OQ613313	OQ613285
*Ap. armeniaca*	SAUCC DL1844	OQ592539	OQ615268	OQ613312	OQ613284
*Ap. arundinis*	CBS 133509	KF144886	KF144930	KF145018	KF144976
*Ap. arundinis*	CBS 44992	KF144887	KF144931	KF145019	KF144977
*Ap. aurea*	CBS 24483 ^T^	AB220251	KF144935	KF145023	KF144981
*Ap. babylonica*	SAUCC DL1841 ^T^	OQ592538	OQ615267	OQ613311	OQ613283
*Ap. babylonica*	SAUCC DL1864	OQ592537	OQ615266	OQ613310	OQ613282
*Ap. bambusicaulis*	GUCC 17.41 ^T^	PP959151	PP959161	PP998074	PP998084
*Ap. bambusicaulis*	GUCC 17.42	PP959152	PP959162	PP998075	PP998085
*Ap. bambusicola*	MFLUCC 20-0144 ^T^	MW173030	MW173087	MW183262	N/A
*Ap. bambusilentiginis*	GUCC 18.51 ^T^	PP959155	PP959165	PP998078	PP998088
*Ap. bambusilentiginis*	GUCC 18.52	PP959156	PP959166	PP998079	PP998089
*Ap. bawanglingensis*	SAUCC BW0444 ^T^	OR739429	OR739570	OR753446	OR757126
*Ap. bawanglingensis*	SAUCC BW0441	OQ592551	OQ615280	OQ613324	OQ613302
*Ap. biserialis*	CGMCC 320135 ^T^	MW481708	MW478885	MW522938	MW522955
*Ap. biserialis*	GZCC 20-0099	MW481709	MW478886	MW522939	MW522956
*Ap. camelliae-sinensis*	LC 5007 ^T^	KY494704	KY494780	KY705103	KY705173
*Ap. camelliae-sinensis*	LC 8181	KY494761	KY494837	KY705157	KY705229
*Ap. cannae*	ZHKUCC 22-0139	OR164902	OR164949	OR166286	OR166322
*Ap. chiangraiense*	MFLUCC 21-0053 ^T^	MZ542520	MZ542524	N/A	MZ546409
*Ap. chromolaenae*	MFLUCC 17-1505 ^T^	MT040106	MT214436	MT235802	N/A
*Ap. cordyline*	GUCC 10026	MT040105	N/A	MT040126	MT040147
*Ap. coryli*	CFCC 58978 ^T^	OR125564	OR133586	OR139974	OR139978
*Ap. coryli*	CFCC 58979	OR125565	OR133587	OR139975	OR139979
*Ap. cyclobalanopsidis*	CGMCC 3.20136 ^T^	MW481713	MW478892	MW522945	MW522962
*Ap. dichotomanthi*	LC 4950 ^T^	KY494697	KY494773	KY705096	KY705167
*Ap. dichotomanthi*	LC 8175	KY494755	KY494831	KY705151	KY705223
*Ap. dongyingensis*	SAUCC 0302 ^T^	OP563375	OP572424	OP573264	OP573270
*Ap. fermenti*	KUC 21289 ^T^	MF615226	N/A	MH544667	MF615231
*Ap. fici*	MFLUCC 19-0156 ^T^	MW114312	MW114392	MW288019	N/A
*Ap. gaoyouensis*	CFCC 52301 ^T^	MH197124	N/A	MH236793	MH236789
*Ap. gaoyouensis*	CFCC 52302	MH197125	N/A	MH236794	MH236790
*Ap. gelatinosa*	KHAS 11962 ^T^	MW481706	MW478888	MW522941	MW522958
*Ap. guizhouensis*	LC 5318	KY494708	KY494784	KY705107	KY705177
*Ap. guizhouensis*	LC 5322 ^T^	KY494709	KY494785	KY705108	KY705178
*Ap. hainanensis*	SAUCC 1681 ^T^	OP563373	OP572422	OP573262	OP573268
*Ap. hispanica*	IMI 326875	AB220243	AB220337	N/A	AB220290
*Ap. hispanica*	IMI 326877 ^T^	AB220242	AB220336	N/A	AB220289
*Ap. hydei*	CBS 114990 ^T^	KF144890	KF144936	KF145024	KF144982
*Ap. hydei*	LC 7103	KY494715	KY494791	KY705114	KY705183
*Ap. hyphopodii*	MFLUCC 15-0003 ^T^	KR069110	N/A	N/A	N/A
*Ap. hyphopodii*	JHB 003	KY356088	KY356093	N/A	N/A
*Ap. hysterina*	ICMP 6889 ^T^	MK014874	MK014841	MK017951	MK017980
*Ap. intestini*	CBS 135835 ^T^	KR011352	KR149063	KR011351	KR011350
*Ap. intestini*	MFLUCC 21-0045	MZ542521	MZ542525	MZ546406	MZ546410
*Ap. italica*	CBS 145138 ^T^	MK014880	MK014847	MK017956	MK017985
*Ap. jatrophae*	AMH-9556	HE981191	N/A	N/A	N/A
*Ap. jatrophae*	AMH-9557 ^T^	NR154675	N/A	N/A	N/A
*Ap. jiangxiensis*	LC 4494	KY494690	KY494766	KY705089	KY705160
*Ap. jiangxiensis*	LC 4577 ^T^	KY494693	KY494769	KY705092	KY705163
*Ap. jinanensis*	SAUCC DL1981 ^T^	OQ592544	OQ615273	OQ613317	OQ613289
*Ap. jinanensis*	SAUCC DL2000	OQ592543	OQ615272	OQ613316	OQ613288
*Ap. koreanum*	KUC 21332 ^T^	MH498524	MH498444	MH544664	MH498482
*Ap. lageniformis*	KUC 21687	ON764023	ON787762	ON806627	ON806637
*Ap. lageniformis*	KUC 21686 ^T^	ON764020	ON787759	ON806624	ON806634
*Ap. locuta-pollinis*	GUCC 10228	MT040124	N/A	MT040145	MT040166
*Ap. locuta-pollinis*	KUNCC 22-12408	OP377736	OP377743	OP381090	N/A
*Ap. locuta-pollinis*	KUNCC 22-12409	OP377737	OP377744	OP381091	N/A
*Ap. locuta-pollinis*	LC 11683 ^T^	MF939595	N/A	MF939616	MF939622
*Ap. longistromum*	MFLUCC 11-0481 ^T^	KU940141	KU863129	N/A	N/A
*Ap. lophatheri*	CFCC 58975 ^T^	OR125566	OR133588	OR139970	OR139980
*Ap. lophatheri*	CFCC 58976	OR125567	OR133589	OR139971	OR139981
*Ap. machili*	SAUCC 1175A-4	OR739433	OR739574	OR753450	OR757130
*Ap. machili*	SAUCC 1175	OQ592560	OQ615289	OQ613333	OQ613307
*Ap. marianiae*	AP 301119	ON692407	ON692423	ON677181	ON677187
*Ap. marii*	CBS 20057	KF144900	KF144946	KF145034	KF144992
*Ap. marii*	CBS 49790 ^T^	AB220252	KF144947	KF145035	KF144993
*Ap. marinum*	KUC 21328 ^T^	MH498538	MH498458	MH544669	MH498496
*Ap. minutisporum*	17E-042 ^T^	LC517882	N/A	LC518889	LC518888
*Ap. montagnei*	AP 301120 ^T^	ON692408	ON692424	ON677182	ON677188
*Ap. mori*	NCYUCC 19-0340	MW114314	MW114394	N/A	N/A
*Ap. mori*	MFLUCC 20-0181 ^T^	MW114313	MW114393	N/A	N/A
*Ap. mukdahanensis*	MFLUCC 22-0056 ^T^	OP377735	OP377742	OP381089	N/A
*Ap. multiloculata*	MFLUCC 21-0023 ^T^	OL873137	OL873138	N/A	OL874718
*Ap. neochinensis*	CFCC 53036 ^T^	MK819291	N/A	MK818545	MK818547
*Ap. neochinensis*	CFCC 53037	MK819292	N/A	MK818546	MK818548
*Ap. neosubglobosa*	JHB 006	KY356089	KY356094	N/A	N/A
*Ap. neosubglobosa*	JHB 007 ^T^	KY356090	KY356095	N/A	N/A
*Ap. obovata*	LC 4940 ^T^	KY494696	KY494772	KY705095	KY705166
*Ap. oenotherae*	CFCC 58972 ^T^	OR125568	OR133590	OR139972	OR139982
*Ap. oenotherae*	LS 395	OR125569	OR133591	OR139973	OR139983
*Ap. ovata*	CBS 115042 ^T^	KF144903	KF144950	KF145037	KF144995
*Ap. paraphaeosperma*	MFLUCC 13-0644 ^T^	KX822128	KX822124	N/A	N/A
*Ap. phragmitis*	CPC 18900 ^T^	KF144909	KF144956	KF145043	KF145001
*Ap. phyllostachydis*	MFLUCC 18-1101 ^T^	MK351842	MH368077	MK340918	MK291949
*Ap. piptatheri*	CBS 145149 ^T^	MK014893	MK014860	MK017969	N/A
*Ap. pseudohyphopodii*	KUC 21680 ^T^	ON764026	ON787765	ON806630	ON806640
*Ap. pseudohyphopodii*	KUC 21684	ON764027	ON787766	ON806631	ON806641
*Ap. pseudoparenchymatica*	LC 7234 ^T^	KY494743	KY494819	KY705139	KY705211
*Ap. pseudoparenchymatica*	LC 8173	KY494753	KY494829	KY705149	KY705221
*Ap. pseudorasikravindrae*	KUMCC 20-0208 ^T^	MT946344	N/A	MT947361	MT947367
*Ap. pseudosinensis*	CPC 21546 ^T^	KF144910	KF144957	KF145044	N/A
*Ap. pseudospegazzinii*	CBS 102052 ^T^	KF144911	KF144958	KF145045	KF145002
*Ap. pterosperma*	CBS 123185	KF144912	KF144959	N/A	KF145003
*Ap. pterosperma*	CPC 20193 ^T^	KF144913	KF144960	KF145046	KF145004
*Ap. pusillisperma*	KUC 21321 ^T^	MH498533	N/A	MN868930	MH498491
*Ap. qinlingensis*	CFCC 52303 ^T^	MH197120	N/A	MH236795	MH236791
*Ap. qinlingensis*	CFCC 52304	MH197121	N/A	MH236796	MH236792
*Ap. rasikravindrae*	NFCCI 2144 ^T^	JF326454	N/A	N/A	N/A
*Ap. sacchari*	CBS 37267	KF144918	KF144964	KF145049	KF145007
*Ap. sacchari*	CBS 66474	KF144919	KF144965	KF145050	KF145008
*Ap. saccharicola*	CBS 191.73	KF144920	KF144966	KF145051	KF145009
*Ap. saccharicola*	CBS 831.71	KF144922	KF144969	KF145054	KF145012
*Ap. sargassi*	KUC 21228 ^T^	KT207746	N/A	MH544677	KT207644
*Ap. septata*	CGMCC 3.20134 ^T^	MW481711	MW478890	MW522943	MW522960
*Ap. serenensis*	IMI 326869 ^T^	AB220250	AB220344	N/A	AB220297
*Ap. setariae*	CFCC 54041 ^T^	MT492004	N/A	MW118456	MT497466
*Ap. sorghi*	URM 93000 ^T^	MK371706	N/A	N/A	MK348526
*Ap. sphaerosperma*	CBS 114314 ^T^	KF144904	KF144951	KF145038	KF144996
*Ap. stipae*	CBS 146804 ^T^	MW883403	MW883798	MW890082	MW890121
*Ap. taeanensis*	KUC 21322 ^T^	MH498515	MH498435	MH544662	MH498473
*Ap. thailandica*	MFLUCC 15-1999	KU940146	KU863134	N/A	N/A
*Ap. thailandica*	MFLUCC 15-0202 ^T^	KU940145	KU863133	N/A	N/A
*Ap. tropica*	MFLUCC 21–0056 ^T^	OK491657	OK491653	N/A	OK560922
*Ap. vietnamensis*	CBS 102053	KF144896	KF144942	KF145030	KF144988
*Ap. vietnamensis*	CBS 251.29	KF144897	KF144943	KF145031	KF144989
*Ap. vietnamensis*	IMI 285638b	AB220241	AB220335	N/A	AB220288
*Ap. vietnamensis*	IMI 99670 ^T^	KX986096	KX986111	N/A	KY019466
*Ap. vietnamensis*	SDBR-CMU 868 = KUNCC 25-21329	PX763177	PX763175	PX761697	PX761699
*Ap. xenocordella*	CBS 59566	KF144926	KF144971	N/A	N/A
*Ap. yunnana*	MFLUCC 15-0002 ^T^	KU940147	KU863135	N/A	N/A
*Nigrospora zimmermanii*	CBS 290.62 ^T^	KY385309	KY806276	KY385311	KY385317
*N. rubi*	LC 2698 ^T^	KX985948	KX986102	KY019302	KY019475

Note: Ex-type strains are marked as “^T^” and newly described strains are indicated in red. The unavailable sequences are indicated as “N/A”.

**Table 2:** Information of fungal taxa used for phylogenetic analyses of *Nigrospora* in this study.

Species Name	Strain Codes	GenBank Accession No.		
		ITS	TUB2	TEF1-α
*Apiospora pseudoparenchymatica*	LC 7234 ^T^	KY494743	KY705211	KY705139
*Ap. vietnamensis*	CBS 102053	KF144896	KF144988	KF145030
*Nigrospora anhuiensis* (nom. inval.)	QY-2	OP677969	PP103614	PP103590
*N. aurantiaca*	CGMCC 3.18130 ^T^	KX986064	KY019465	KY019295
*N. aurantiaca*	LC 7034	KX986093	KY019598	KY019394
*N. bambusae*	CGMCC 3.18327 ^T^	KY385307	KY385319	KY385313
*N. bambusae*	LC 7245	KY385305	KY385321	KY385315
*N. brasiliensis*	CMM 1214 ^T^	KY569629	MK720816	MK753271
*N. brasiliensis*	CMM 1217	KY569630	MK720817	MK753272
*N. camelliae-sinensis*	CGMCC 3.18125 ^T^ = LC 3500	KX985986	KY019460	KY019293
*N. camelliae-sinensis*	LC 6304	KX986045	KY019566	KY019370
*N. chinensis*	LC 6851	KX986049	KY019579	KY019450
*N. chinensis*	CGMCC 3.18127 ^T^	KX986023	KY019462	KY019422
*N. cooperae*	SFC 20230324-M03	OQ726361	OQ735179	OQ735196
*N. cooperae*	BRIP 72408b	OP035047	OP039537	OP039538
*N. coryli* (nom. inval.)	W18	PP218065	PP320372	PP461302
*N. covidalis*	CGMCC 3.20538 ^T^	OK335209	OK431479	OK431485
*N. covidalis*	LC 158337	OK335210	OK431480	OK431486
*N. dactylidis*	KUNCC 25-19190 ^T^	PV608638	PV613324	N/A
*N. dactylidis*	KUNCC 25-19191	PV608639	PV613325	N/A
*N. dicranopteridis*	KUNCC 23–14199 ^T^	PV138724	PV222011	PV177116
*N. endophytica*	URM 8712 = A.R.M. 687	OM265226	OP572418	OP572415
*N. endophytica*	URM 8462 = A.R.M. 973 ^T^	OM265233	OP572420	OP572416
*N. falsivesicularis*	CGMCC 3.19678 ^T^	MN215778	MN329942	MN264017
*N. falsivesicularis*	LC 13553	MN215779	MN329943	MN264018
*N. ficuum*	ZHKUCC 22-0143 ^T^	OR164911	OR166318	N/A
*N. globosa*	CGMCC 3.19633 ^T^	MK329121	MK336134	N/A
*N. globosa*	LC 12441	MK329122	MK336135	N/A
*N. globospora*	CGMCC 3.20539 ^T^	OK335211	OK431481	OK431487
*N. globospora*	LC 15839	OK335212	OK431482	OK431488
*N. gorlenkoana*	CBS 480.73 ^T^	KX986048	KY019456	KY019420
*N. guangdongensis*	CFCC 53917 ^T^	MT017509	MT024495	MT024493
*N. guangdongensis*	Tly 068	MT017510	MT024496	MT024494
*N. guangxiensis*	KUNCC 23-16747 ^T^	PQ553688	PQ613610	PQ613605
*N. guilinensis*	LC 7301	KX986063	KY019608	KY019404
*N. guilinensis*	CGMCC 3.18124 ^T^	KX985983	KY019459	KY019292
*N. hainanensis*	CGMCC 3.18129 ^T^	KX986091	KY019464	KY019415
*N. hainanensis*	URM 8714 = A.R.M. 967	OM265228	OM793057	OM642834
*N. humicola*	CFCC 56884 ^T^	ON555686	ON557392	ON557394
*N. humicola*	CFCC 56885	ON555687	ON557393	ON557395
*N. humicola*	MFG 70052	OK563251	OK626373	OK626389
*N. iteae*	SDBR-CMU 867 = KUNCC 25-21328 ^T^	PX763176	PX761699	PX761697
*N. lacticolonia*	CGMCC 3.18123 ^T^	KX985978	KY019458	KY019291
*N. lacticolonia*	URM 8713 = A.R.M. 921	OM265227	OM642838	OM642833
*N. macarangae*	ZHKUCC 23-0003 ^T^	PP091035	PP646185	PP646182
*N. magnoliae*	MFLUCC 19–0112 ^T^	MW285092	MW438334	N/A
*N. magnoliae*	LC 6704	KX986047	KY019571	KY019373
*N. manihoticola*	URM 8461 = A.R.M. 645 ^T^	OM265224	OM869479	OM914791
*N. marylouisemclawsiae*	BRIP 74865b ^T^	PP125567	PP209362	PP209361
*N. musae*	CBS 319.34 ^T^	KX986076	KY019455	KY019419
*N. musae*	LC 6385	KX986042	KY019567	KY019371
*N. oryzae*	LC 2724	KX985959	KY019486	KY019312
*N. oryzae*	LC 4265	KX985994	KY019518	KY019335
*N. osmanthi*	CGMCC 3.18126 ^T^	KX986010	KY019461	KY019421
*N. osmanthi*	LC 4487	KX986017	KY019540	KY019438
*N. pernambucoensis*	URM 8711 = A.R.M. 651	OM265225	OM869480	OM914792
*N. pernambucoensis*	URM 8463 = A.R.M. 974 ^T^	OM265234	OM869481	OM914793
*N. philosophiae-doctoris*	CGMCC 3.20540 ^T^	OK335213	OK431483	OK431489
*N. philosophiae-doctoris*	LC 15838	OK335214	OK431484	OK431490
*N. platycladiensis*	CFCC 72632 ^T^	PV759478	PV855114	PV820528
*N. platycladiensis*	CFCC 72630	PV759479	PV855115	PV820529
*N. pubeiensis*	KUNCC 23-16745 ^T^	PQ553686	PQ613608	PQ613603
*N. pyriformis*	CGMCC 3.18122 ^T^	KX985940	KY019457	KY019290
*N. pyriformis*	URM 8716 = A.R.M. 970	OM265231	OM642839	OM513904
*N. rubi*	LC 2698 ^T^	KX985948	KY019475	KY019302
*N. saccharicola*	LC 12057	MN215789	MN329952	MN264028
*N. saccharicola*	CGMCC 3.19362 ^T^	MN215788	MN329951	MN264027
*N. sacchari-offcinarum*	CGMCC 3.19335 ^T^	MN215791	MN329954	MN264030
*N. sacchari-offcinarum*	LC 13531	MN215792	MN329955	MN264031
*N. shadeganensis*	IRAN 4958C = SCUA-Saf-N27 ^T^	PP256499	PP263821	PP263812
*N. shadeganensis*	SCUA-Saf-N28	PP256500	PP263822	PP263813
*N. singularis*	CGMCC 3.19334 ^T^	MN215793	MN329956	MN264032
*N. singularis*	LC 12068	MN215794	MN329957	MN264033
*N. sphaerica*	LC 2839	KX985964	KY019491	KY019317
*N. sphaerica*	LC 2840	KX985965	KY019492	KY019318
*N. stoneae*	BRIP 75022a	OR608744	OR604067	OR604065
*N. tomentosae*	ZHKUCC 22-0339 ^T^	PP759659	PP763296	PP763294
*N. tomentosae*	ZHKUCC 22-0340	PP759660	PP763297	PP763295
*N. vesicularifera*	CGMCC 3.19333 ^T^	MN215812	MN329975	MN264051
*N. vesicularifera*	URM 8718 = A.R.M. 975	OM265235	OM642840	OM513905
*N. vesicularis*	LC 0322	KX985939	KY019467	KY019296
*N. vesicularis*	CGMCC 3.18128 ^T^	KX986088	KY019463	KY019294
*N. wurfbainiae* (replaced syn. *N. guangdongense*)	ZHKUCC 24–0545 ^T^	PV523285	PV536733	PV536735
*N. wurfbainiae* (replaced syn. *N. guangdongense*)	ZHKUCC 24–0546	PV523286	PV536734	PV536736
*N. yunnanensis*	GUCC 24-0008 ^T^	PP915796	PP947937	PP947933
*N. yunnanensis*	GUCC 24-0009	PP915797	PP947938	PP947934
*N. zimmermanii*	CBS 290.62 ^T^	KY385309	KY385317	KY385311
*N. zimmermanii*	CBS 984.69	KY385310	KY385322	KY385316

Note: Ex-type strains are marked as “^T^” and newly described strains are indicated in red. The unavailable sequences are indicated as “N/A”.

**Table 3:** Morphological comparison between the new strain (SDBR-CMU868) and closely related strains.

Species	Hosts/Substrates	Conidiophores	Conidiogenous Cells	Conidia	Sterile Cells	References
***Ap. vietnamensis* (SDBR-CMU868)**	**Sporulation in vitro (PDA) isolated from the healthy stem of *Itea riparia***	**Reduced to conidiogenous cells**	**Aggregated in clusters on hyphae, basauxic, hyaline to pale brown, doliiform to ampulliform or clavate, 3.5–7 × 2.5–4 μm**	**Pale brown to brown, smooth, globose to subglobose in surface view, lenticular in side view, with a pale equatorial slit, 5–7 × 4–6 μm**	**Brown, elongated ellipsoidal to clavate, branched, 58–15 × 2.5–3.5 μm**	**This study**
*Ap. vietnamensis* (IMI 99670, ex-type)	Sporulation in vitro (PDA), isolated from decayed fruit of *Citrus sinensis*	Lateral hyphal branches or short, branched structures, 4.5–7.5 µm thick	ND	Aggregated, pale brown to dark brown, globose, 5–6 μm in surface view, 3–4 μm in side view	ND	[5,35]
*Ap. vietnamensis* (CBS 102053) (=*Ap. malaysiana*)	Sporulation in vitro (PDA) isolated from the *Macaranga hullettii* stem colonized by ants	Reduced to conidiogenous cells	Aggregated in clusters on hyphae, basauxic, hyaline to pale brown, doliiform to clavate to ampulliform, 4–7 × 3–5 µm	Brown, smooth, globose in surface view, lenticular in side view, with a pale equatorial slit, 5–6 µm in surface view, 3–4 µm in side view	ND	[1]
*Ap. vietnamensis* (IMI 285638b) (=*Ap. euphorbiae*)	Dead stems of *Euphorbia* sp.	Erect or ascending, simple, flexuous, cylindrical, colorless, except for the numerous brown transverse septa, 15–110 × 0.5–1 µm	ND	Brown or olivaceous brown, smooth, 4–5.5 μm in surface view, 3–4 μm in side view	Hemispherical, 4–5 × 2–3 μm	[1,36]

Note: Newly generated isolate is indicated by black bold. ND = not determined.

**Table 4:** A checklist of *Nigrospora* species with relevant important data.

Species Name	Asexual Morphological Features	Cultures Media ^a^	Molecular Data ^b^	Ecological Roles ^c^	Habitats and Hosts ^d^	Geographical Distribution ^e^	Biological Activities	Literature Source
*Nigrospora aerophila*	NA	NA	–	NA	Air	South America (Brazil)	NA	[54]
*N. anhuiensis* *	Conidia globose or oval, 11.25–15.75 µm	Carrot medium	+	P	*Oryza sativa*	Asia (Anhui Province, China)	NA	[50]
*N. arundinacea* (basionym: *Hadrotrichum arundinaceum*)	Conidiophores reduced to conidiogenous cells; conidiogenous cells pale brown, subglobose or ampulliform; conidia black, globose or subglobose, solitary, 17–21 μm diam.	NA	–	S	*Arundo conspicua*	Europe (England)	NA	[5,55]
*N. aurantiaca*	Conidiophores reduced to conidiogenous cells; conidiogenous cells pale brown, ovoid or ampulliform; conidia black, ellipsoidal, solitary, 12–16.5 × 9–15.5 μm	PDA with orange pigment	+	P, E	***Nelumbo* sp.**, *Castanea mollissima*, *Nicotiana tabacum*, *Musa paradisiaca*, *Saccharum officinarum*	Asia (Jiangxi and Hainan Provinces, China, and Thailand)	Potential pigment for textile dyeing, anti-cancer, anti-inflammatory	[5,56,57]
*N. bambusae*	Conidiophores reduced to conidiogenous cells; conidiogenous cells pale brown, subglobose to ampulliform; conidia black, globose or subglobose, solitary, 13.5–17.5 × 10–17 μm	PDA	+	P, E	*Bambusoideae*	Asia (Guangdong and Jiangxi Provinces China)	NA	[5]
*N. brasiliensis*	Conidiophores reduced to conidiogenous cells; conidiogenous cells pale brown to dark brown, doliiform, ampulliform, globose with hyaline vesicles; conidia black, ovoid, subglobose or globose, solitary, 15.6 × 28.6 μm	PDA	+	P	*Nopalea cochenillifera*	South America (Brazil)	NA	[58]
*N. camelliae-sinensis*	Conidiophores reduced to conidiogenous cells; conidiogenous cells hyaline to pale brown, globose to ampulliform or clavate; conidia black, globose or ellipsoid, solitary, 12–18 × 9–14.5 μm	PDA	+	P, E	***Camellia sinensis***, *Musa paradisiaca*, *Castanopsis* sp., *Saccharum officinarum*	Asia (Guangxi, Hainan and Yunnan Provinces, China)	NA	[5]
*N. canescens*	NA	NA	–	P, E	*Musa* sp.	Oceania (Australia)	NA	[59]
*N. chinensis*	Conidiophores reduced to conidiogenous cells; conidiogenous cells hyaline, ampulliform or subglobose; conidia black, globose or subglobose, solitary, 10–14 μm diam.; sterile cells pale to dark brown, elongated ellipsoidal to clavate	PDA	+	P, E	***Machilus breviflora***, *Camellia sinensis*, *Castanopsis* sp., *Itea* sp., *Lindera aggregata*, *Machilus* sp., *Musa paradisiaca*, *Osmanthus* sp., *Quercus* sp., *Smilax ocreata*	Asia (Jiangxi, Guangxi, and Yunnan Provinces, China)	Anti-bacterial, anti-fungal	[5,60,61]
*N. cooperae*	Conidiophores reduced to conidiogenous cells; conidiogenous cells pale brown, doliiform to ampulliform to subglobose; conidia black, ellipsoidal, solitary, 10–12.5 × 8.5–10.5 μm	PDA, SNA, OA	+	P	*Heteropogon contortus*	Oceania (Australia)	NA	[62]
*N. coryli* *	Conidiophores reduced to conidiogenous cells; conidiogenous cells pale brown, globose to subglobose; conidia black, globose, ellipsoidal, solitary, 13.5–17.5 × 11.5–14 μm	PDA	+	E	*Corylus heterophylla*	Asia (Guizhou Province, China)	NA	[49]
*N. covidalis*	Conidiophores hyaline to pale brown, flexuous or straight; conidiogenous cells pale brown, doliiform to ampulliform; conidia pale brown to black, globose, sparse, 9–14 μm diam.	PDA, SNA	+	P	*Lithocarpus* sp.	Asia (Jiangxi Province, China)	NA	[63]
*N. dactylidis*	Conidiophores pale brown, straight, septate, conidiogenous cells hyaline, cylindrical, conidia pale brown to dark brown, globose to subglobose, 15–19 × 11–19 μm diam.	PDA	+	S	*Dactylis glomerata*	Asia (Yunnan Province, China)	NA	[64]
*N. dicranopteridis*	Conidiophores reduced to conidiogenous cells; conidiogenous cells hyaline to dark brown, subspherical or ampulliform; conidia black, globose to subglobose, sparse, 10–13 × 10–11 μm.; chlamydospore pale brown to dark brown, clavate, obclavate	PDA, SNA	+	E	*Dicranopteris ampla*	Asia (Guizhou Province, China)	NA	[65]
*N. endophytica*	Conidiophores reduced to conidiogenous cells; conidiogenous cells pale brown to dark brown, globose; conidia pale brown to dark brown, globose to subglobose, solitary, 10–17.5 μm diam.	PDA	+	E	*Manihot esculenta*	South America (Brazil)	NA	[15]
*N. falsivesicularis*	Conidiophores pale brown, flexuous or straight; conidiogenous cells pale brown, doliiform to ampulliform; conidia pale brown to black, globose or subglobose, sparse, 9–16 μm diam.	PDA	+	P	*Saccharum officinarum*	Asia (Guangxi Province, China)	NA	[66]
*N. ficuum*	Conidiophores pale brown, straight, conidiogenous cells hyaline, cylindrical, conidia dark brown, globose to subglobose, with a longitudinal germ-slit, 8–12 × 10–12 μm	PDA	+	S	*Ficus* sp.	Asia (Guangdong Province, China)	NA	[67]
*N. gallarum* (basionym: *Basisporium gallarum*, current name: *N. oryzae*)	NA	NA	–	NA	NA	NA	NA	[68]
*N. globosa*	Conidiophores reduced to conidiogenous cells; conidiogenous cells hyaline to pale brown, cylindrical, ampulliform, ellipsoidal or subglobose; conidia dark brown to black, subglobose to globose, solitary, 11.0–14.5 × 9.0–13.0 μm	PDA	+	S	soil in cave	Asia (Guangxi Province, China)	NA	[69]
*N. globospora*	Conidiophores reduced to conidiogenous cells; conidiogenous cells, subspherical or ampulliform; conidia black, ellipsoidal, sparse, 8.5–12 × 10.5–13.5 μm	PDA, SNA	+	P	*Petasites hybridus*	Asia (Fujian Province, China)	NA	[63]
*N. gorlenkoana*	Conidiophores pale brown; conidiogenous cells pale brown, doliiform to ampulliform; conidia pale brown to black, globose or subglobose, solitary, 11.5–17 μm diam.	PDA	+	P, E	***Vitis vinifera***, *Cirsium setosum*, *Cucumis melo*	Asia (Shandong Province, China, and Kazakhstan)	NA	[5]
*N. gossypii* (current name: *N. oryzae*)	NA	NA	–	NA	***Gossypium* sp.**, *Abies alba*	Europe (Poland), Asia (Russia, and Uzbekistan)	NA	[70]
*N. guangdongensis*	Conidiophores reduced to conidiogenous cells; conidiogenous cells hyaline or pale brown, doliiform to ampulliform; conidia dark brown to black, globose or subglobose, solitary, 13.65–20.9 μm	PDA	+	P	*Cunninghamia lanceolata*	Asia (Guangdong Province, China)	NA	[40]
*N. guangxiensis*	Conidiophores reduced to conidiogenous cells; conidiogenous cells pale brown to brown, subglobose; conidia dark brown to black, subglobose to globose, solitary, 8–14 μm diam.	PDA	+	E	*Aquilaria sinensis*	Asia (Guangxi Province, China)	NA	[23]
*N. guilinensis*	Conidiophores reduced to conidiogenous cells; conidiogenous cells hyaline, doliiform to clavate to ampulliform; conidia black, globose or subglobose, solitary, 11.5–15 μm diam.	PDA	+	P, E	***Camellia sinensis***, *Nelumbo* sp., *Phellodendron chinense*	Asia (Guangxi Province, China)	Anti-fungal	[5,71]
*N. hainanensis*	Conidiophores reduced to conidiogenous cells; conidiogenous cells hyaline, globose or ampulliform; conidia black, globose or ellipsoidal, solitary, 12.5–17.5 μm diam.; setae straight to irregularly curved, black, subcylindrical	PDA	+	P, E	***Musa paradisiaca***, *Nopalea cochenillifera*, *Saccharum officinarum*	Asia (Hainan Province, China), South America (Brazil)	NA	[5]
*N. humicola*	Conidiophores hyaline to brown; conidiogenous cells pale brown, subglobose to ampulliform; conidia black, globose to subglobose, solitary, 12.5–23.5 × 9.5–16 µm	PDA	+	S	**Soil**, *Phragmites australis*	Asia (Hebei Province, China, and Russia)	NA	[7,22]
*N. iteae*	Conidiophores brown, septate, straight or flexuous; conidiogenous cells brown, ampulliform to clavate; conidia black, globose to subglobose, solitary, 15.5–21.5 × 12.5–19 µm	PDA with pale orange pigment	+	E	*Itea japonica*	Asia (Thailand)	NA	This study
*N. javanica*	NA	NA	–	NA	NA	NA	NA	[72]
*N. lacticolonia*	Conidiophores reduced to conidiogenous cells, conidiogenous cells pale brown, finely verruculose, globose to clavate to doliiform, conidia black, globose or ellipsoid, solitary, 11.5–16.5 μm diam.	PDA, SNA	+	P, E	***Camellia sinensis***, *Hylocereus polyrhizus*, *Musa paradisiaca*, *Saccharum officinarum*	Asia (Jiangxi, Hainan, Guangxi, Guangdong Provinces, China, and Malaysia)	Anti-phytopathogenic, plant growth-promoting, insecticidal	[5,66,73]
*N. macarangae*	Conidiophores reduced to conidiogenous cells; conidiogenous cells pale brown, globose to subglobose to ampulliform; conidia dark brown to black, globose or subglobose, solitary, 14.5 × 16 μm	PDA	+	S	*Macaranga tanarius*	Asia (Taiwan, China)	NA	[32]
*N. magnoliae*	Conidiophores hyaline, flexuous or straight; conidiogenous cells hyaline, doliiform to ampulliform; conidia dark brown, globose or subglobose, solitary, 10–14 × 10–13 μm diam.	PDA	+	E	*Magnolia candollei*	Asia (Yunnan Province, China)	NA	[74]
*N. manihoticola*	Conidiophores reduced to conidiogenous cells; conidiogenous cells pale brown to dark brown, globose to subglobose to ovoid; conidia dark brown to black, globose to subglobose to ellipsoidal, solitary, 10–17.5 μm diam.	PDA	+	E	*Manihot esculenta*	South America (Brazil)	NA	[15]
*N. marylouisemclawsiae*	NA	NA	+	NA	*Spinifex sericeus*	Oceania (Australia)	NA	[52]
*N. maydis* (basionym: *Sporotrichum maydis*)	NA	NA	–	NA	NA	NA	NA	[75]
*N. mercuriadeae*	NA	NA	+	P	*Chromolaena odorata*	Oceania (Australia)	NA	[53]
*N. musae*	Conidiophores pale brown, flexuous or straight; conidiogenous cells pale brown, subglobose to ampulliform with hyaline vesicle; conidia black, globose or subglobose, solitary, 15–19.5 μm diam.	PDA, SNA	+	P, E	***Musa* sp.**, *Camellia sinensis*,	Oceania (Australia)	NA	[5,59]
*N. neosacchaicola*	Conidiophores reduced to conidiogenous cells; conidiogenous cells pale brown, subglobose to pot-shape; conidia brown to black, globose or subglobose, solitary, 11.4–16.7 × 8.1–13.4 μm	CMA, MEA, OA, PDA, SNA, WA	+	P	*Juglans regia*	Asia (Yunnan Province, China)	NA	[38]
*N. oryzae* (basionym: *Monotospora oryzae*)	Conidiophores pale brown, flexuous or straight; conidiogenous cells hyaline, ampulliform or subspherical; conidia black, globose or subglobose, solitary, 12.5–16 μm diam.	PDA, SNA	+	P, E	***Oryza sativa***, various plants, mostly Poaceae	Africa, Asia, North America, Central America, South America, Europe, Oceania	Anti-fungal, phytotoxic, anti-malarial, anti-trypanosome, neuroprotective, plant growth-promoting	[5,76,77,78,79]
*N. osmanthi*	Conidiophores reduced to conidiogenous cells; conidiogenous cells hyaline to pale brown, ampulliform to cylindrical; conidia black, globose, solitary, 13.5–16.5 μm diam.	PDA, SNA	+	P, E	***Osmanthus* sp.**, *Hedera nepalensis*, and other various hosts	Asia (Jiangxi Province, China, and Malaysia)	NA	[5]
*N*. *padwickii*	NA	NA	–	NA	*Oryza sativa*	Asia (India)	NA	[80]
*N. panici*	Conidiophore short, simple, subhyaline; conidia subglobose, 25–30 × 22–25 μm	NA	–	S	*Panicum amphibium*	Oceania (Australia), Asia (Hongkong Province, China, and Indonesia)	NA	[10,81]
*N. pernambucoensis*	Conidiophores reduced to conidiogenous cells; conidiogenous cells pale brown to dark brown, globose to obpyriform with hyaline vesicles; conidia pale or dark brown to black, globose or ellipsoidal, solitary, 12.5–20 μm diam.	PDA	+	E	*Manihot esculenta*	South America (Brazil)	NA	[15]
*N. philosophiae-doctoris*	Conidiophores reduced to conidiogenous cells; conidiogenous cells pale brown, subglobose to ampulliform; conidia pale brown to brown, globose or subglobose, sparse, 11–16 × 8–14 μm	PDA, SNA	+	P	*Disporum sessile*	Asia (Guangxi, China)	NA	[63]
*N. platycladi* (as *‘platycladiensis’*)	Conidiophores reduced to conidiogenous cells; conidiogenous cells, hyaline to pale brown, ampulliform to subcylindrical; conidia brown to black, subglobose, solitary, 10.4–17.5 × 9.7–17.3 μm	PDA	+	P	*Platycladus orientalis*	Asia (Beijing, China)	NA	[48]
*N. pubeiensis*	Conidiophores hyaline to pale gray, subcylindrical; conidiogenous cells hyaline or dark brown, subglobose; conidia dark brown to black, subglobose or globose, solitary, 6–14 μm	PDA	+	E	*Aquilaria sinensis*	Asia (Guangxi Province, China)	NA	[23]
*N. pyriformis*	Conidiophores reduced to conidiogenous cells; conidiogenous cells pale brown, ampulliform or subcylindrical; conidia pale brown to black, dimorphic, globose, 12.5–16.5 μm diam., or pyriform, 17.5–27.5 × 10–18.5 μm	PDA, SNA	+	P, E	***Citrus reticulata***, *Musa paradisiaca*, *Camellia sinensis*, *Lindera aggregate*, *Rosa* sp., *Rubus reflexus*, *Castanopsis* sp.	Asia (Jiangxi and Hainan, Province, China)	NA	[5]
*N. rubi*	Conidiophores reduced to conidiogenous cells; conidiogenous cells pale brown, subglobose to ampulliform to lageniform; conidia black, globose, solitary, 11.5–16.5 μm	PDA, SNA	+	P, E	***Rubus* sp.**, *Fraxinus* sp.	Asia (Jiangxi Province, China)	NA	[5]
*N. sacchari* (basionym: *Glenospora sacchari*)	NA	NA	+	P, E	*Saccharum officinarum*, Poaceae and other hosts	Africa, Asia, South America, Oceania	Phytotoxic	[82,83]
*N. saccharicola*	Conidiophores reduced to conidiogenous cells; conidiogenous cells hyaline to pale brown, clavate or globose to ampulliform; conidia black, globose, solitary, 13.5–16.5 μm diam., sterile cells elongated, intermingled among conidia	PDA	+	P	*Saccharum officinarum*	Asia (Guangxi Province, China)	NA	[66]
*N. sacchari-officinarum*	Conidiophores reduced to conidiogenous cells; conidiogenous cells pyriform or ampulliform, 0–1 septate, with hyaline vesicles; conidia black, globose or subglobose, solitary, 14.5–19.5 μm diam.	PDA	+	P	*Saccharum officinarum*	Asia (Guangxi Province, China)	NA	[66]
*N. shadeganensis*	Conidiophores pale brown, flexous; conidiogenous cells hyaline to pale brown, subspherical to cylindrical; conidia dark brown to black, globose or subglobose to ellipsoidal, solitary, 11.5–17.5 μm diam.	PDA	+	E	*Halocnemum strobilaceum*	Asia (Iran)	NA	[84]
*N. singularis*	Conidiophores reduced to conidiogenous cells; conidiogenous cells ovoid or ampulliform; conidia black, ellipsoid to subglobose, solitary, 9.5–13 × 11–15 μm	PDA	+	P	*Saccharum officinarum*	Asia (Guangdong Province, China)	NA	[66]
*N. solani*	Conidiophores reduced to conidiogenous cells; conidiogenous cells ampulliform; conidia black, globose to subglobose, solitary, 12–14 × 15 μm	PDA, SNA	+	E	*Solanum lycopersicum* var. *cerasiforme*	South America (Brazil)	NA	[85]
*N. sphaerica* (basionym: *Trichosporum sphaericum*, current name: *Nigrospora oryzae*)	Conidiophores hyaline to pale brown, multiseptate, extensively branched; conidiogenous cells pale brown, subglobose; conidia black, globose or subglobose, 16–21 μm diam.	PDA, SNA	+	P, E	*Zea mays* and various hosts	Africa, Asia, North America, Central America, South America, Oceania	Anti-fungal anti-bacterial, cytotoxic, anti-inflammatory, enzyme inhibition, antimicrobial, antioxidant, and cytotoxic	[5,82,86,87,88,89,90,91]
*N. stoneae*	NA	NA	+	P	*Cyperus aromaticus*	Oceania (Australia)	NA	[51]
*N. tomentosae*	Conidiophores reduced to conidiogenous cells; conidiogenous cells hyaline to pale brown, globose to pot-shaped or clavate; conidia dark brown to black, globose or subglobose, solitary, 12–17 × 12–17 μm; chlamydospore pale brown, subglobose to globose	PDA, SNA	+	E	*Citrus grandis* cv. *Tomentosa*	Asia (Guangdong Province, China)	NA	[92]
*N. vesicularifera*	Conidiophores reduced to conidiogenous cells; conidiogenous cells pale brown, short-ampulliform, with hyaline vesicles; conidia black, globose, solitary, 11–19 μm diam.	PDA	+	P	*Saccharum officinarum*	Asia (Guangdong Province, China)	NA	[66]
*N. vesicularis*	Conidiophores reduced to conidiogenous cells; conidiogenous cells hyaline to pale brown, doliiform to ampulliform, with hyaline vesicles; conidia solitary, black, globose or ellipsoidal, 12.5–16.5 × 9–15 μm	PDA, SNA	+	P, E	*Musa paradisiaca*	Asia (Hainan Province, China)	NA	[5]
*N. vietnamensis* (current name: *Apiospora vietnamensis*)	Conidia brown, aggregate, globose, 5–6 μm diam.	PDA	+	S	*Citrus sinensis*	Europe (Czech Republic)	NA	[5]
*N. wurfbainiae* (replaced synnonym: *N. guangdongensis* (as ‘*guangdongense*’))	Conidiophores reduced to conidiogenous cells; conidiogenous cells hyaline, globose to subglobose to ampulliform; conidia dark brown to black, subglobose to globose, solitary, 10–15 × 4–15 μm	PDA	+	E	*Wurfbainia villosa*	Asia (Guangdong Province, China)	NA	[39]
*N. yunnanensis*	Conidiophores hyaline to brown; conidiogenous cells pale brown, subglobose to ampulliform; conidia black, globose to subglobose, solitary, 14.5–18.5 × 11–17.5 µm	PDA, OA	+	P	*Juglans regia*	Asia (Yunnan, China)	NA	[47]
*N. zimmermanii*	Conidiophores hyaline to pale brown; conidiogenous cells hyaline to pale brown, ampulliform; conidia dark brown, globose or ellipsoid, solitary, 11–18 × 14–18 µm	PDA, SNA	+	P, E	*Saccharum officinarum*	Asia (China), South America (Ecuador)	Anti-cancer, anti-inflammatory	[5,93,94]

Note: “NA” represented no data available. ^a^ The culture media given are potato dextrose agar (PDA), synthetic nutrient-poor agar medium (SNA), oatmeal agar (OA). ^b^ Molecular data are marked as available sequence data (+) and unavailable sequence data (−) based on the GenBank database (https://www.ncbi.nlm.nih.gov/, accessed on 1 November 2025). ^c^ Ecological roles are given as pathogen (P), endophyte (E), and saprobe (S). ^d^ Habitats and hosts: when the species occurred on many hosts, the first host was indicated in bold. ^e^ Habitats, hosts, and geographical distribution data were extracted from the USDA Fungus-Host databases (https://fungi.ars.usda.gov/, assessed on 1 November 2025), Index Fungorum (https://indexfungorum.org/Names/Names.asp, assessed on 20 March 2026). * The species names are marked with the nomenclatural comments: Nom. inval., Art. 40.1 (Shenzhen).

## Data Availability

All sequences generated in this study were submitted to GenBank (https://submit.ncbi.nlm.nih.gov/, accessed on 1 November 2025).
